# Genome-Environment Association Analysis for Bio-Climatic Variables in Common Bean (*Phaseolus vulgaris* L.) from Brazil

**DOI:** 10.3390/plants10081572

**Published:** 2021-07-30

**Authors:** Júlio Cesar F. Elias, Maria Celeste Gonçalves-Vidigal, Andrea Ariani, Giseli Valentini, Maria da Conceição Martiniano-Souza, Mariana Vaz Bisneta, Paul Gepts

**Affiliations:** 1 Departamento de Agronomia, Universidade Estadual de Maringá-UEM, Av. Colombo 5790, Maringá 87020-900, Brazil; juliocesar_net@hotmail.com (J.C.F.E.); mariamartiniano@gmail.com (M.d.C.M.-S.); marianavazbisneta@hotmail.com (M.V.B.); 2 BASF BBCC-Innovation Center, 9052 Gent, Belgium; andrea_ariani@yahoo.it; 3 Soybean Genomics and Improvement Laboratory USDA-ARS, 10300 Baltimore Avenue, Beltsville, MD 20705, USA; Giseli.Valentini@usda.gov; 4 Department of Plant Sciences, Section of Crop and Ecosystem Sciences, University of California, Davis, CA 95161-8780, USA; plgepts@ucdavis.edu

**Keywords:** genome-wide association study (GWAS), single nucleotide polymorphism (SNP), regional adaptation, water stress

## Abstract

Abiotic stress is a limiting factor for common bean (*Phaseolus vulgaris L.*) production globally. The study of the genotypic, phenotypic, and bio-climatic variables in a broad set of accessions may assist the identification of genomic regions involved in the climatic adaptation of the common bean. We conducted a genotyping-by-sequencing analysis using 28,823 SNPs on 110 georeferenced common bean accessions from Brazil to discover associations between SNPs and bio-climatic indexes. The population structure analysis clustered the accessions into two groups corresponding to the Andean and Mesoamerican gene pools. Of the 19 bioclimatic variables, 17 exhibited a significant association with SNPs on chromosomes Pv01, Pv02, Pv03, Pv04, Pv06, Pv09, Pv10, and Pv11 of common bean. Ten candidate genes were associated with specific bio-climatic variables related to temperature and precipitation. The candidate genes associated with this significant Pv09 region encode a Platz transcription factor family protein previously reported to be an essential regulator of drought stress. The SNP markers and candidate genes associated with the bio-climatic variables should be validated in segregating populations for water stress, which could further be used for marker-assisted selection. As a result, bean breeding programs may be able to provide advances in obtaining drought-tolerant cultivars.

## 1. Introduction

Common bean (*Phaseolus vulgaris* L.) is one of the most important legume crops, providing 15% of the total daily calories and 36% of the complete daily protein in parts of Africa and the Americas [1,2,3,4]. Beans are considered to be a primary source of protein in several countries, particularly those that fall below the poverty line [5,6]. Interestingly, Brazil is one of the world’s leading producers [7] and consumers of *P*. *vulgaris*. Common bean production in Brazil is challenging because of the wide range of different climates, soils, cultivars, and levels of agricultural technology [8].

Currently, climate change is considered to be a significant concern. Agriculture is one of the activities that is most affected by climate change, either by variations in annual rainfall, heatwaves, average temperature, or global increases in atmospheric CO_2_ or ozone levels [9]. Thus, climate change will have consequences for food security due to the reduction in agricultural production [8]. Bean production systems are no exception to climate change impacts. Rainfall shortages are the main abiotic factor that directly affects common bean grain yield, affecting approximately 60% of global production [10]. In this sense, cultivar adaptation is the most effective strategy for reducing the vulnerability of common bean and other crops to climate change [8].

The identification of drought tolerance traits is a prerequisite for developing molecular tools that help breeding programs, such as marker-assisted selection. Several studies identified QTLs associated with drought tolerance [11,12,13,14,15,16,17], but few exhibited stable expression in different environments, which restricted their use in research and breeding conditions.

The duration, intensity, and rate of progression can characterize a specific type of drought stress. Usually, drought is enhanced by the occurrence of other factors that also cause stress, such as temperature, luminosity, diseases, pests, and nutrient-poor soils [11,12]. Identifying QTLs for drought-related traits is limited to the almost exclusive use of the mapped population, and particularly biparental mapping, which allows the investigation of a few alleles for one of the targeted genes. Several alleles may exist, and association analysis is a means to discover the locations of these genes, understand the feasibility of the identified QTLs, and implement them in breeding programs using marker-assisted selection [18,19,20].

Association analysis is frequently a method of choice to detect genotypic–phenotypic associations due to the accessibility of highly multiplexed molecular tools, such as whole-genome resequencing [21,22,23], using populations and germplasm accessions. This approach is feasible because of linkage disequilibrium at two or more loci, resulting in the nonrandom association of alleles. When two loci are in complete linkage disequilibrium, alleles can be used to predict those in the other locus. The recombination between two loci depends on multiple factors, such as the genomic region, population size, selection method, and genetic drift, which, in turn, affect the local recombination rate, nonrandom mating, mutation rate, and population structure [24,25,26,27]. Germplasm bank collections with accessions from diverse origins, including accessions such as landraces (sometimes called “crioulo” cultivars in Brazil), have significant advantages because they harbor genetic diversity at different levels of linkage disequilibrium.

Several studies have described specific traits related to drought and their potential use in breeding programs to identify more tolerant genotypes [14,28,29]. In this context, Beebe [14] conducted a retrospective review of the different characteristics in common bean that are related to drought tolerance. Drought tolerance QTLs can be identified through association studies via aspects related to plant physiology within the crop.

Moreover, studies have discovered that genome–environment associations (GEAs) can potentially predict factors involved in abiotic stress adaptation in plants. In this study, we sought to identify genome-wide single nucleotide polymorphisms (SNPs) that are associated with climate parameters at the accessions’ sampling site [28]. 

The assumption is that genome–environment associations reflect the adaptation of common bean to abiotic stresses at the sampling sites. This approach can be used to efficiently identify loci that control drought tolerance in bean germplasm [28,29]. For example, Cortés and Blair [28] found 115 SNPs in 90 regions, which were widespread in all 11 common bean chromosomes and associated with a bio-climatic-based drought index. López-Hernández and Cortés [29] identified 120 SNPs associated with different bioclimatic variables, and five bean accessions (G2648, G23511A, G13094, G12869, and G11071) were assumed to be tolerant to the stress. Ariani et al. [30] evaluated 246 accessions of wild *Phaseolus vulgaris* that were related to distribution and associated environmental changes. These authors identified five subpopulations distributed in different dry forests and observed distinct distributions of temperature and rainfall, resulting in decreased local potential evapotranspiration.

In the present study, we evaluated common bean accessions from smallholder farmers settled in different Brazilian regions. These accessions are cultivated annually and are exposed to adverse climatic conditions, such as high temperature and rainfall shortages during the critical growth phase, such as flowering and pod fill. We characterized these common bean genotypes using genotyping-by-sequencing, evaluated their genetic structure, and identified QTLs associated with 19 bio-climatic variables obtained from the WorldClim database.

## 2. Results

### 2.1. Genotyping by Sequencing (GBS) Analysis

A total of 180,515 SNPs identified in 175 common bean accessions were used for imputation, and were processed and analyzed using NGSEP software. Markers with more than 95% absence data and an allelic frequency <0.05% were excluded. After this filtering process, 28,823 SNPs identified in 110 bean accessions were used for association mapping. The markers were distributed on all 11 common bean chromosomes (Table 1, Figure S1).

The SNP distribution across the 11 chromosomes showed an average of 2620 SNPs (57 SNPs per Megabase—Mb). Chromosomes Pv09 (*n* = 3310 SNPs) and Pv02 (*n* = 3651 SNPs) exhibited the highest number of SNPs, whereas Pv10 (*n* = 1660 SNPs) and Pv07 (*n* = 2266 SNPs) showed the fewest. Patterns of nucleotide substitution via the GBS technique classified SNPs as either transition (Ts) or transversions (Tv) (Table 2). We observed that the number of transitions (17,186) was higher than the number of transversions (11,637). Among the transversions, the most frequent was the A/T type (3646). The Ts/Tv ratio was 1.58.

### 2.2. Genetic and Population Structure

**Figure 1:**
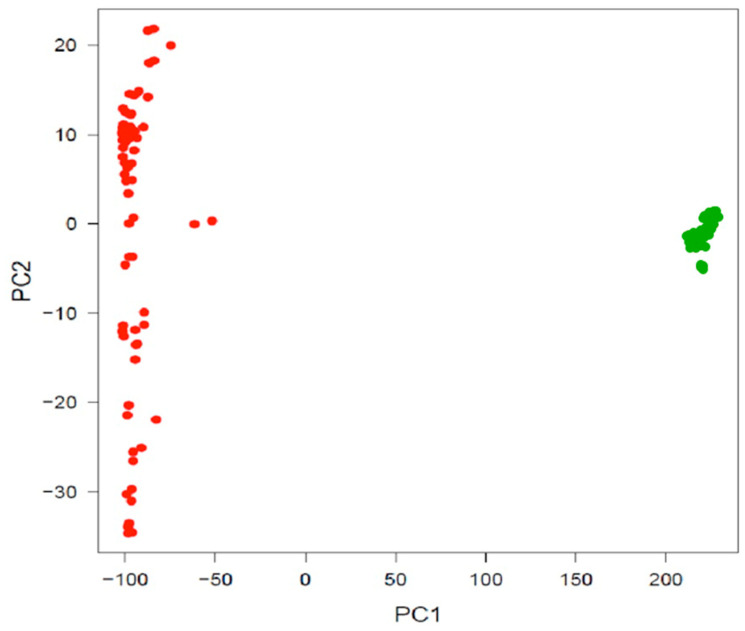
Principal component analysis (PCA) for the 110 common bean accessions from Brazil, using 28,823 SNPs markers.

Principal component analysis (PCA) data revealed that the accessions were clustered into two distinct groups, which corresponded to either the Andean (33 accessions) or Mesoamerican (77 accessions) gene pool (Figure 1). The first (PC1) principal component explained 80% of the variation among accessions and separated the Mesoamerican and Andean accessions. The second principal component explained 0.006% of the total variation. It was responsible for distinguishing the Mesoamerican accessions, which were more diverse than the Andean accessions, as shown by the high dispersion of the points on the two-dimensional plane (Figure 1).

**Figure 2:**
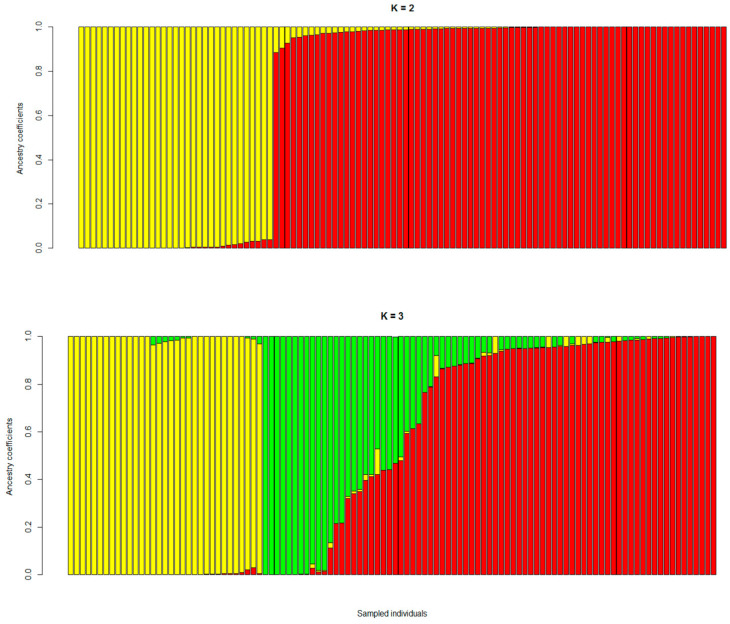
Bar plot for population structure of the 110 common bean accessions. Samples were sorted in the same order and classified conforming to successive selected *K* values from 2 to 10 subpopulations. The groups were identified for *K = 2* and *K = 3*.

Based on K = 2 groups, the population structure analysis showed that the common bean accessions were distributed between two gene pools, Andean and Mesoamerican (Figure 2; Figure S2), with a low degree of admixture. By comparison, when considering K = 3 groups, the Andean accessions were still clustered in a unique subpopulation, Group 1. In contrast, the Mesoamerican accessions formed several subpopulations with considerable admixture, and it was not possible to distinguish these based on market class seed-type. Similarly, results in genetic diversity studies in common bean were obtained using GBS analysis [30], SSR, and SCAR markers [31], and nucleotide sequences [2].

**Figure 3:**
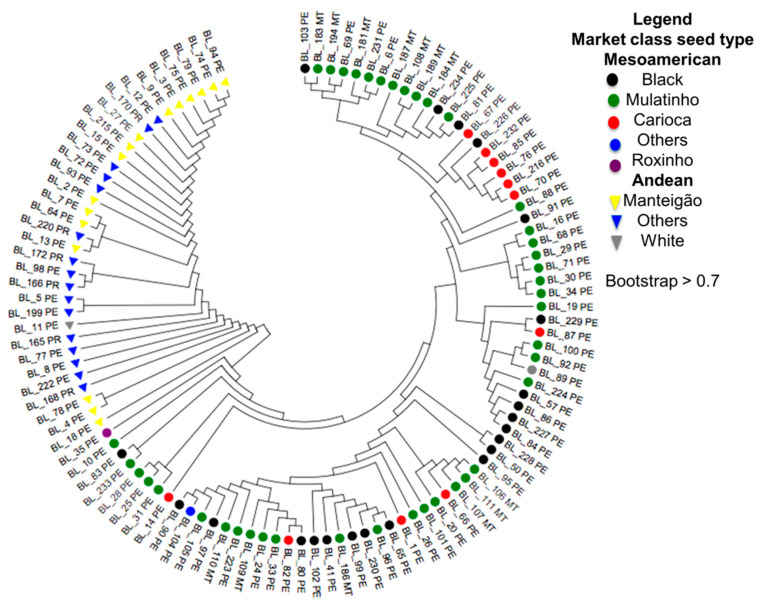
Phylogenetic tree of 110 common bean accessions genotyped with 28,823 SNPs. The color of each accession is based on different market class seed type.

The results of the phylogenetic analysis for the 110 common bean accessions genotyped through GBS were composed of two large groups, one of them Andean and the other Mesoamerican (Figure 3). The clustering agrees with that obtained by the principal component and population structure analyses. The most remarkable genetic diversity was observed among accessions classified as Mesoamerican based on a membership index of >0.7. Within the Mesoamerican gene pool, the accessions were classified according to the commercial group. It can be verified that the accessions from the Mulatinho market class represent a complex group with high genetic diversity because they share alleles with other commercial groups, such as Preto and Carioca. Most individuals were grouped into a specific subpopulation, and some accessions presented mixtures between subpopulations.

### 2.3. Genome-Environment Association Study (GEA)

**Figure 4:**
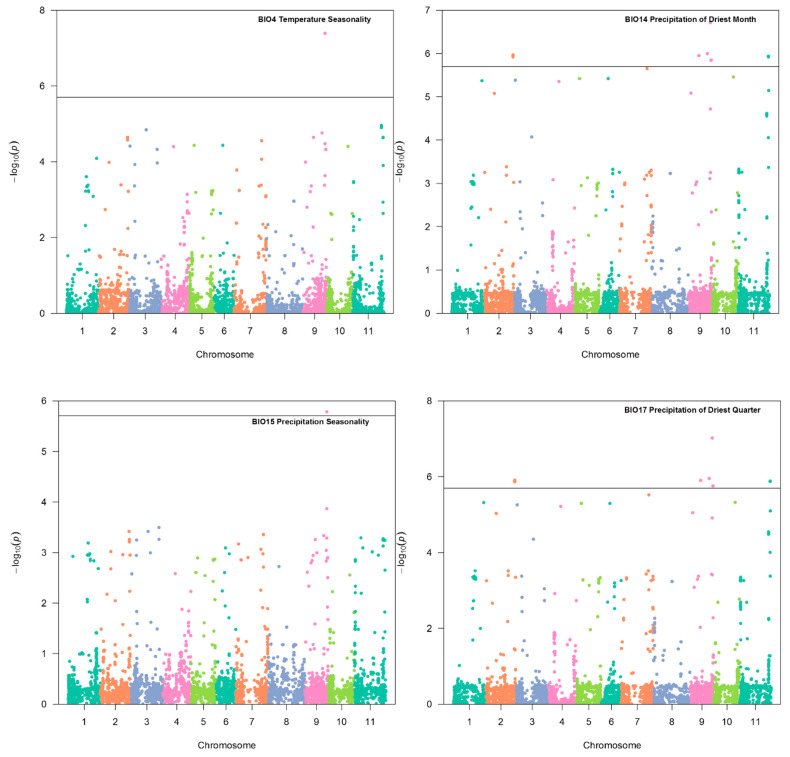
Genome–environment association analysis in common bean accessions. Manhattan plot showing candidate single nucleotide polymorphisms and −log10 (*p*-values) for bio-climatic variables BIO4, BIO14, BIO15, and BIO17. The horizontal black line corresponds to the Bonferroni significance threshold (1.73 × 10^−6^ for α = 0.05 and 28,823 SNP markers).

**Figure 5:**
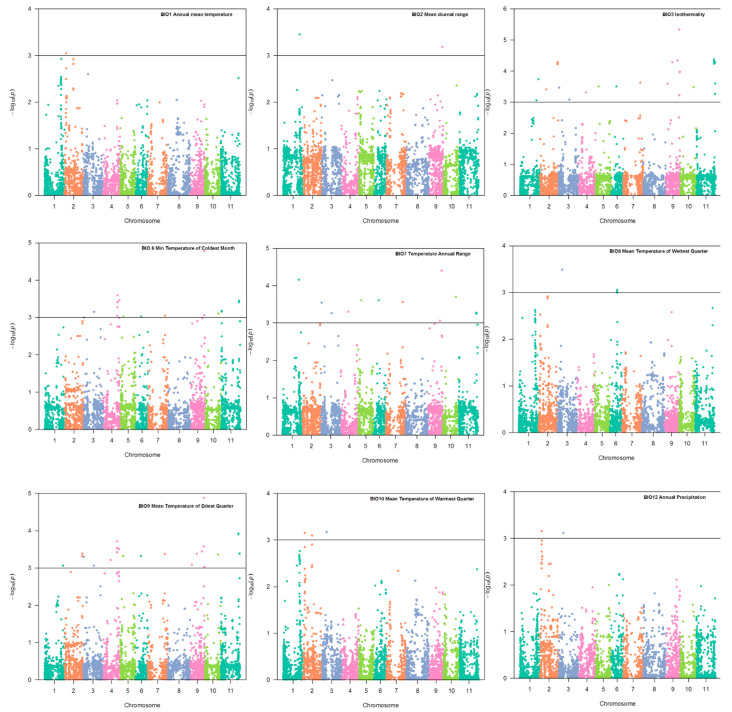
Genome–environment association analysis in common bean accessions. Manhattan plot showing candidate single nucleotide polymorphisms and −log10 (*p*-values) for bio-climatic variables BIO1, BIO2, BIO3, BIO6, BIO7, BIO8, BIO9, BIO10, and BIO12. The horizontal black line corresponds to the significance threshold with *p*-value equal to or smaller than 0.001%.

**Figure 6:**
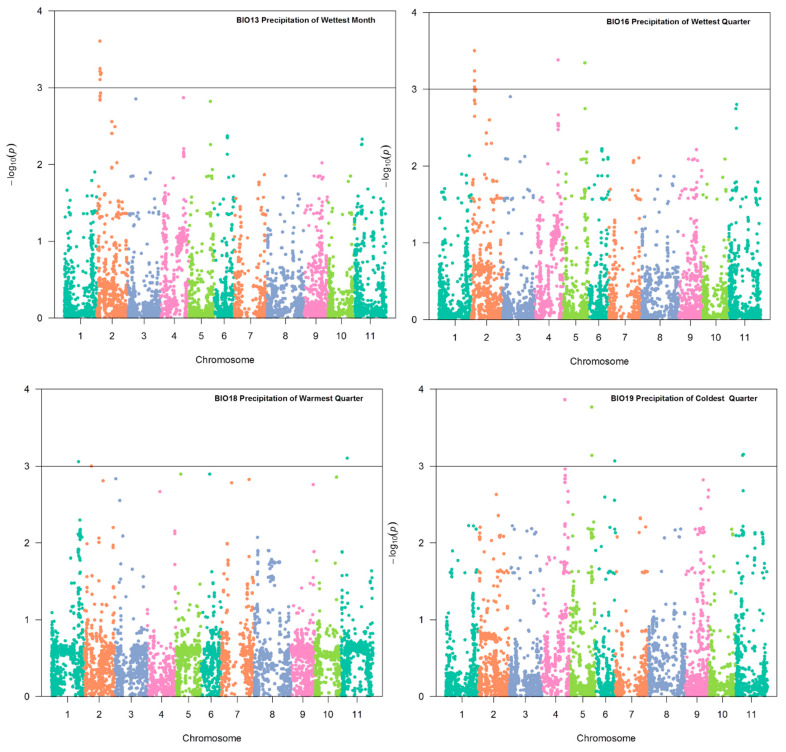
Genome–environment association analysis in common bean accessions. Manhattan plot showing candidate single nucleotide polymorphisms and −log10 (*p*-values) for bio-climatic variables BIO13, BIO16, BIO18, and BIO19. The horizontal black line corresponds to the significance threshold with *p*-value equal to or smaller than 0.001%.

The model (Q + K) provided a better fit for all climatic variables in association analysis. Of the 19 bio-climatic variables evaluated, 17 exhibited a significant association with SNPs distributed on chromosomes Pv01, Pv02, Pv03, Pv04, Pv06, Pv09, Pv10, and Pv11 of common bean. Only the variables BIO5 and BIO11 did not reveal significant associations. The genomic regions associated with the variables temperature seasonality (BIO4), precipitation of driest month (BIO14), precipitation seasonality (BIO15), and precipitation of driest quarter (BIO17) were selected with a significance greater than that established by the Bonferroni correction (1.73 × 10^−6^ for α = 0.05 and 28,823 SNP markers). The SNPs associated with these variables reside on chromosomes Pv02 and Pv09 (Figure 4). The variables BIO1, BIO2, BIO3, BIO6, BIO7, BIO8, BIO9, BIO10, and BIO12 (Figure 5), in addition to BIO13, BIO16, BIO18, and BIO19 (in Figure 6), showed significant SNPs with a *p*-value <0.001%. Table 3 shows the two most considerable SNP markers associated with each variable. All significant SNPs with a *p*-value < 0.001% are included in Table S1.

#### 2.3.1. Chromosome Pv01

Our data revealed the presence of one SNP marker on Pv01 at position 43,225,566 bp, which was associated with BIO2 (mean diurnal range, *p*-value = 3.5 × 10^−4^), BIO7 (temperature annual range, *p* = 6.9 × 10^−5^), and BIO18 (precipitation of warmest quarter, *p* = 8.7 × 10^−4^) (Figure 5 and Figure 6). This marker explained 13.5, 16.6, and 13.0% of the phenotypic variation in BIO2, BIO7, and BIO18, respectively. This SNP is close to the gene model Phvul.001G169300, which encodes a cation/H (+) antiporter 20.

#### 2.3.2. Chromosome Pv02

Table 3 shows two SNPs positioned at 4,770,785 bp and 5,007,350 bp, spanning a genomic region of 237 kb on chromosome Pv02. The significant SNP S02_4770785 was associated with the bio-climatic variables BIO1 (annual mean temperature, *p* = 8.96 × 10^−4^), BIO10—mean temperature of warmest quarter (*p* = 7.02 × 10^−4^), BIO13 (precipitation of wet month, *p* = 2.47 × 10^−4^), and BIO16 (precipitation of wettest quarter, *p* = 3.15 × 10^−4^), as shown in Figure 5 and Figure 6. This marker explained 11.7, 12.6, 17.7, and 13.9% of the phenotypic variation of BIO1, BIO10, BIO13, and BIO16, respectively. The S02_4770785 marker is located within the unannotated Phvul.002G051700 gene model. The S02_5007350 marker was associated with BIO12 (*p* = 6.98 × 10^−4^) and BIO13 (*p* = 5.63 × 10^−4^), and explained 11.3 and 13.6% of the variation, respectively (Figure 5 and Figure 6). This marker is located close to the gene model Phvul.002G055050, which encodes a DNAJ heat shock *n*-terminal-domain-containing protein (Table 3).

#### 2.3.3. Chromosome Pv03

As shown in Figure 6, the GEA results indicated that the Pv03 S03_13038972 SNP marker is associated with BIO8 (mean temperature of wettest quarter, *p* = 3.24 ×10^−4^), BIO10 (mean temperature of warmest quarter, *p* = 6.72 × 10^−4^), and BIO12 (annual precipitation, *p*-value = 7.68 × 10^−4^). Furthermore, the phenotypic variation explained by this SNP was 13.6, 12.7, and 13.1%, respectively. The S03_13038972 marker was close to the Phvul.003G081100 gene model on Pv03, which encodes a protein with no annotation.

#### 2.3.4. Chromosome Pv04

GEA identified two significant SNPs positioned at 36,754,502 bp and 36,771,335 bp, spanning a genomic region of 16.8 kb on chromosome Pv04. SNP S04_36754502 was associated with BIO 16 (precipitation of wettest quarter, *p* = 4.15 × 10^−4^) and BIO 19 (precipitation of coldest quarter, *p* = 1.37 × 10^−4^), and explained 14.5 and 14.0% of the phenotypic variation, respectively (Figure 6). S04_36771335 was associated with bio-climatic BIO6 (*p* = 1.37 × 10^−4^) and variable BIO19 (*p* = 2.56 × 10^−4^) (Figure 5 and Figure 6). This SNP explained 13.5 and 14.0% of the phenotypic variation, respectively. These two SNPs are close to candidate gene Phvul.004G111600, which encodes a solute carrier family with 13 members.

#### 2.3.5. Chromosome Pv06

On chromosome Pv06, the SNP located at position 18,985,703 was associated with a response to BIO8 (mean temperature of wettest quarter, *p* = 8.76 × 10^−4^), which explains 11.1% of the phenotypic variation (Figure 6). This SNP is close to the gene model Phvul.006G071300, which encodes a basic leucine zipper region (bZIP_2). Furthermore, bZIPs are one of the largest families of transcription factors that play diverse roles in plant growth and development, including the regulation of cellular responses in plants under stress conditions.

#### 2.3.6. Chromosome Pv09

This study identified two significant SNPs on Pv09 spanning a region of 4.8 Mb between positions 30,671,474 bp and 35,475,381 bp. The S09_30671474 SNP was associated with BIO14 (precipitation of driest month, *p* = 1.1 × 10^−6^) and BIO17 (precipitation of driest quarter, *p* = 1.11 × 10^−6^) (Figure 4). This SNP explained 25.7 and 25.4% of the phenotypic variation, respectively. This SNP was close to the gene model Phvul.009G207800, which encodes leucine-rich repeat (LRR) proteins, some of which contain an F-box. Interestingly, the other SNP identified on chromosome Pv09, S09_35475381, was significantly associated with nine of the nineteen bio-climatic variables: BIO2, BIO3, BIO4, BIO6, BIO7, BIO9, BIO14, BIO15, and BIO17 (Figure 4 and Figure 5). This SNP explained 11.9 to 36.6% of the phenotypic variation in the mentioned bio-climatic variables. This SNP is close to the candidate gene Phvul.009G241300, which encodes a PLATZ transcription factor protein (Wang et al. 2018) family.

#### 2.3.7. Chromosome Pv11

The SNP located at the beginning of chromosome Pv11 at position 9167,649 was associated with the response of common bean to BIO18 (precipitation of warmest quarter, *p* = 7.86 × 10^−4^) (Figure 6), which explained 10.9% of the phenotypic variation. The gene model Phvul.011G091600, located close to this SNP, encodes NTKL-binding protein 1. In addition, the SNP situated at the end of chromosome Pv11, at position 45,868,325, was associated with common bean responses to BIO 3 (isothermality (*p* = 4.20 × 10^−5^), BIO 4 (temperature seasonality, *p* = 1.10 × 10^−5^), and BIO 9 (mean temperature of driest quarter, *p* = 1.18 × 10^−4^) (Figure 4 and Figure 5), which explained 16.2, 21.1, and 15.5% of the phenotypic variation, respectively.

## 3. Discussion

Genome–environment association studies were performed to identify genomic loci that are associated with climatic traits and to define the potential genetic architecture of these associations, assuming that it is the result of genetic adaptation to the respective climatic traits of the locations or regions of origin. In addition to increasing our understanding of the genetics of adaptation, this approach could be useful in developing an effective, climate-stress-oriented breeding strategy via marker-assisted selection [32]. Nevertheless, high-throughput and reliable genotyping and phenotyping techniques that are used in this approach demand significant effort. The GBS method used in this study is a reliable, inexpensive, simple, and efficient method for high-throughput genotyping [21,33].

GBS analysis based on the reference genome of *P. vulgaris* [1] allowed the identification of a total of 28,823 SNPs in 110 highly divergent common bean accessions from Brazil. We found SNPs on all chromosomes, but they were not evenly distributed. Many of them were in peritelomeric regions (gene-rich regions), whereas fewer SNPs were in pericentromeric parts. Not surprisingly, we observed a lower density of mapped reads and SNPs in these regions. Studies conducted by Ariani [22,30], using GBS analysis based on the *Cvi*AII enzyme, showed the power of this enzyme in detecting genetic divergence among common bean accessions pools.

Our results show that the common bean accessions in our sample included members of the Andean and Mesoamerican gene pools. In addition, subdivisions within these groups were observed, confirming previously published work [1,34,35,36,37,38]. The K = 3 approach did not reveal further subdivisions among accessions of Andean origin. In contrast, the same method showed the presence of two subdivisions and admixture between Mesoamerican accessions. Burle et al. [39] investigated the genetic diversity and structural population of common bean accessions from Brazil using microsatellites. The authors obtained similar results but identified nine subpopulations.

Admixture is a characteristic feature of the genetic structure among Brazilian bean accessions, especially within the Mesoamerican gene pool. The current results confirm those of Burle et al. [39] and, subsequently, those of Blair et al. [40]. Because the Mesoamerican gene pool of Brazilian common bean displays substantial introgression, there could be less structure in this gene pool, which would favor the application of genome-wide association, especially for traits such as bio-climatic variables differentiating the accessions of our sample. Knowledge of plant geographical distribution and genetic diversity is crucial for collecting, protecting, and monitoring genetic resources. In addition, genetic diversity is influenced by both geographical distribution and environmental conditions [41]. These ecological variables appear to play a significant role in differentiating populations through selection pressure and local adaptation [42].

If associations between single-nucleotide polymorphism (SNP) alleles and the environment of origin in crop landraces reflect adaptation, these could be used to predict phenotypic variation for adaptive traits [42]. The average performance across environments can be essential to identifying genotypes with superior performance despite the spatial-temporal and environmental heterogeneity present in the field. According to Lasky et al. [42], ecological associations were good predictors of average phenotypes. For example, in sorghum, the per-plant biomass trait was predicted by SNPs with a strong association with aridity, where genotypes with alleles associated with arid environments exhibited higher biomass.

Previous studies have identified an association between SNPs and significant bio-climatic variables such as temperature and precipitation [42,43]. Associations among bioclimatic traits and the distribution of Andean and Mesoamerican subpopulations indicates that the Mesoamerican group is subjected to an environment with higher precipitation instability because it is located in tropical regions with high rain indexes. In contrast, the Andean group endures stable rain indexes and is at higher altitudes [30]. Regarding seasonal temperatures, accessions from the Mesoamerican group exhibited a trend to withstand higher temperature variations than accessions from the Andean group [30]. All of these significant variations in targeted bio-climatic traits demonstrate an adaptation of common bean accessions to continuous environmental changes. Wild *P. vulgaris* is situated in Neotropical seasonally dry forest areas, one of the most threatened biomes of the world [44]. Brazilian regions such as the Northeast region, including Pernambuco state, where the majority of the accessions of the current sample were collected, exhibit similar environmental characteristics to those of this biome. This region constantly experiences a lack of rain and a high temperature throughout the year.

The Mulatinho type of beans has seeds that are dark cream and almost brown in color; they are Mesoamerican, small-seeded beans, which are more widely accepted in the Northeast region of Brazil [40], an area characterized by severe periods without rain and high temperatures. Consequently, cultivars from the Mulatinho group can exhibit high yield stability when subjected to soil water deficit [45].

In the present study, 11 novel SNPs were significantly associated with common bean responses to 17 bio-climatic variables. These SNPs were located on chromosomes Pv01, Pv02, Pv03, Pv04, Pv06, Pv09, and Pv11. We searched for candidate genes close to these SNPs at NCBI (National Center for Biotechnology Information; https://www.ncbi.nlm.nih.gov, accessed on 20 June 2021), and functional annotation was verified in the Phytozome database. The SNP S01_43225566 associated with the traits at Pv01 is close to the gene model Phvul.001G169300, which encodes a cation/H(+) antiporter 20. Cation/H+ antiporters are essential regulators of intracellular ion homeostasis and are critical for cell expansion and plant stress acclimation [46]. In Arabidopsis, cation/H+ exchanger proteins (AtCHX) mediate K+ transport and pH homeostasis and are localized in the intracellular and plasma membranes [47]. Thus, the CHX genes in plants imply a significant role of ion and pH homeostasis in dynamic endomembranes of vegetative and reproductive tissues [48]. In Arabidopsis, this gene is expressed in leaves and roots, especially in dermal tissues, such as the root epidermis [49], root cap, and guard cells [50]. Diaz et al. [51] mapped the QTL Yd1.1 for seed yield under drought conditions in the chromosome Pv01 located in a different position, i.e., 11,250,640bp, from the region identified in the present study. Trapp et al. [16] validated the QTL SY1.1BR for seed yield under drought conditions in the chromosome Pv01, located 47.7 Mb closer to the marker SNP50809. Interestingly, this QTL is located 4.5 Mb from the S01_43225566 marker found to be associated with BIO2, BIO7, and BIO18 in the present study.

A previous study [16] reported QTL for seed yield under drought conditions in the chromosome Pv02, the QTL SY2.1BR located 11.8 Mb closer to the marker SNP40055. The S02_4770785 marker identified in the present study at Pv02 is located 7.1Mb from the QTL previously identified. Furthermore, S02_4770785 is close to Phvul.002G055050, which encodes a DNAJ heat shock *n*-terminal domain-containing protein. Heat shock proteins (HSPs) help plants cope with adverse conditions caused by stresses such as drought, salt stress, exposure to chemicals, and exposure to highly variable temperature (heat and cold) stress [52]. HSPs help to maintain cellular homeostasis by maintaining the three-dimensional structure of cellular proteins. In addition, the presence of high- to low-molecular-weight HSP classes in the plant genome points toward a precisely evolved adaptation mechanism. Furthermore, using sense transgenic Arabidopsis plants that were constitutively expressing elevated levels of DnaJ, Zhichang et al. [53] observed that overexpression of DnaJ could confer NaCl stress tolerance.

GEA results identified the S03_13038972 SNP marker in Pv03 associated with BIO8, BIO10, and BIO12. This SNP is located in a different position from previously reported QTLs. Mukeshimana et al. [15] found the QTL SW3.1SC in the chromosome Pv03 associated with seed weight under drought conditions using RILs from the cross SEA5/CAL96. The markers ss715646941 and ss715649325 are close to SW3.1SC and are located at 2,620,445 and 3,670,201, respectively. The marker ss715646941 was also detected in QTL analysis for seed yield under drought stress using the 20 most drought-tolerant and 20 most susceptible RILS from the cross Buster × Roza [16].

SNPs S04_36754502 and S04_36771335 were close to the candidate gene Phvul.004G111600, which encodes a solute carrier family of 13 members. This gene model is a homolog of an Arabidopsis thaliana divalent ion symporter. AtNIP5;1 (Arabidopsis) and OsNIP2;1 (rice) are located in the plasma membrane in plants and perform physiologically significant roles in the uptake of the nutritionally essential metalloids boron and silicon, respectively [54].

The SNP S06_18985703 is close to the gene model Phvul.006G071300, which encodes a basic region leucine zipper (bZIP_2). bZIPs are among the most prominent families of transcription factors, which play diverse roles in plant growth development, including regulating cellular responses in plants under stress conditions [55]. The authors observed highly upregulated expression of EcbZIP60 under drought-, osmotic-, salt-, and methyl viologen-induced stress in finger millet. Transgenic Tabaco plants containing bZIP cloned from finger millet showed improved tolerance to drought stress with higher stomatal conductance and photosynthesis, resulting in improved growth. In potato, genome-wide identification analysis coupled with RNA-Seq expression data led to the identification of candidate StbZIPs that are dysregulated and could play a pivotal role under various abiotic stress conditions [56].

SNP S09_35475381 was close to the candidate gene Phvul.009G241300, which encodes a PLATZ transcription factor (TF) protein. PLATZ is a plant AT-rich sequence and involves zinc-binding proteins that constitute a plant-specific transcription factor family with two conserved zinc-dependent DNA-binding domains. The PLATZ proteins perform significant functions in regulating plant development and resistance. *Brassica* PLATZ genes vary, including in tissue-specific, stress-responsive, and hormone-responsive expression [57]. TFs are essential regulators of drought stress. Comparative transcriptome analysis in maize by Zenda et al. [58] observed that the PLATZ-TF7 gene was upregulated in the drought-tolerant line YE8112. Mukeshimana et al. [15] found the QTL SY9.2SC in the chromosome Pv09 was associated with seed yield under drought conditions. This QTL is linked to the SNP marker ss715640302 at 31,677,655 bp, about 1.0 Mb from the QTL identified in this study.

The SNP S09_30671474 was close to the gene model Phvul.009G207800, which encodes a leucine-rich repeat (LRR) protein; some of these proteins contain an F-box. The leucine-rich repeat (LRR)-containing domain is well known to act in innate immunity in plants, serving as the first line of defense against biotic stresses. LRRs also promote interactions between LRR proteins, as observed in receptor–coreceptor complexes [59]. However, using global genome expression profiling of rice subjected to cold, drought, or heat stresses, Liao et al. [60] observed that leucine-rich repeat receptor-like kinase 2 was highly upregulated under cold and drought stress. According to Song et al. [61], F-box proteins play critical roles in selective and specific protein degradation through the 26S proteasome. These authors observed that most F-box protein genes could respond to salt and heavy metal stresses using microarray analysis. Using real-time PCR analysis, these authors confirmed that some of the F-box protein genes containing heat-, drought-, salicylic acid-, and abscisic acid-responsive cis-elements could respond to abiotic stresses in *Medicago truncatula*.

The gene model Phvul.011G091600 located close to SNP S11_9167649 encodes NTKL-binding protein 1. Limited information is available regarding the function of this protein; however, it is known to act as a transcriptional activator, playing a role in the stress response in plants. Based on real-time quantitative PCR analyses, He et al. [62] showed that the expression of this gene was affected by salt, drought, cold, ABA, and other stress conditions in Arabidopsis. In addition, the overexpression of this gene in wild-type Arabidopsis resulted in increased salt tolerance compared with wild-type plants.

Aiming to identify loci putatively involved in common bean environmental adaptation, Ariani and Gepts [63] searched for genes associated with specific bio-climatic variables related to temperature and precipitation. A total of 49 genes located in all 11 reference genome chromosomes, except for Pv06, were associated with the 18 bio-climatic variables. Several of these encode proteins related to hormone response, ion homeostasis, plant development, metabolism, and stress response, in particular drought. Because the authors studied wild beans, they identified different candidate genes. For example, the authors identified Phvul.001G034400, a homolog of Arabidopsis KEA6 involved in potassium homeostasis; Phvul.010G155000, involved in ABA signaling; Phvul.010G035200, a homolog of a cytokinin responsive factor homologous of Arabidopsis; and Phvul.008G161700, homologous to an Arabidopsis thioredoxin involved in ROS signaling.

In the current study, whole-genome genotyping of 110 common bean accessions from Brazil resulted in wide genetic diversity compared to previous studies. In addition, the association between genetic and geoecological data allowed the identification of 11 SNPs significantly associated with common bean response to 17 bio-climatic variables. These SNPs are located on chromosomes Pv01, Pv02, Pv03, Pv04, Pv06, Pv09, and Pv11. Furthermore, we identified candidate genes encoding different proteins for each SNP. Of particular note is SNP S09_35475381 on chromosome Pv09, which was associated with nine of the nineteen bio-climatic variables. This SNP is linked to the candidate gene model Phvul.009G241300, which encodes a PLATZ transcription factor family protein previously known to be an essential regulator of drought stress. The SNP markers and gene models identified should be validated, and may efficiently reduce the cost and valuable time required in common bean breeding programs using marker-assisted selection.

According to Singh [64], traits related to water use, such as drought tolerance and water deficit, can be identified, especially in Mesoamerican accessions. Most recently, Onziga et al. [65] described QTLs for drought tolerance in Andean bean germplasm, which was previously reported only in Mesoamerican germplasm. Consequently, these QTLs could be used to improve drought tolerance in Andean beans.

Variations in specific bio-climatic traits, such as the mean minimum temperature of the coldest month and seasonal temperature, favor Andean accessions because they are more adapted to regions of mesic temperature, which is also associated with intermediate altitudes [22]. The variation in these bio-climatic variables between gene pools could determine the increase in ecological amplitude (amplitude increase/bio-climatic variation of each region) and adaptation potential to continuous climatic changes. Therefore, the presence of genetic diversity in the Andean and Mesoamerican gene pools of *P. vulgaris* and other species related to *Phaseolus* is essential to guarantee the adaptation of common bean to environments that do not fit those previously described [17,66]. Moreover, the drought tolerance of wild beans consists of their more vital ability when compared with domesticated types to continue growth despite water-limited conditions [67]. These authors were the first to report bean responses to drought in an environment of origin for a diverse selection of wild beans.

## 4. Material and Methods

### 4.1. Plant Materials

A total of 110 common bean accessions collected in Brazilian rural communities located in the states of Pernambuco (*n* = 86), Paraíba (*n* = 3), Sergipe (*n* = 2), Mato Grosso (*n* = 13), and Paraná (*n* = 6) were investigated (Table 4, Figure S5). Seed propagation and DNA preparation was conducted at the Núcleo de Pesquisa Aplicada à Agricultura—Nupagri Laboratory (Universidade Estadual de Maringá, Maringá, Brazil).

### 4.2. DNA Isolation and Library Preparation for Sequencing

Genomic DNA isolation and library preparation were performed following the GBS method based on the methylation-insensitive restriction enzyme *Cvi*AII [22]. DNA quality and quantity assessment were determined by NanoDrop Lite (Thermo Fisher Scientific, Waltham, USA) and electrophoresis (1% agarose gel). DNA samples that exhibited an absorbance ratio (A260/A280) >1.7 and no visible degradation on agarose gel were further used for library preparation. A QUBIT dsDNA HS assay kit was used to quantify genomic DNA and library adapters. Because *Cvi*AII was selected to perform the sequencing protocol, specific barcodes and adapters for this restriction enzyme were designed with a GBS barcode adapter generator (http://www.deenabio.com/services/gbs-adapters, accessed on 30 June 2021) (Table S2). Sample multiplexing consisted of two libraries with 110 accessions in each. A blank sample and the *P. vulgaris* genotype (G19833) were used as quality controls in each of the two libraries.

The Experion DNA analysis kit (Bio-Rad, Berkeley, CA, USA) verified the presence of adapter dimer contamination in sequencing libraries. A total of two genomic libraries were sequenced using the Illumina HiSeq4000 platform to generate 50 bp single-end reads using the QB3 Vincent J. Coates Genomics Sequencing Laboratory at the University of California, Berkeley, USA. Raw sequencing reads are available in the NCBI Sequence Read Archive.

### 4.3. Sequence Alignment and SNP Calling

The sequence read alignment and SNP calling steps were conducted as previously described [22]. For read alignment, we used the reference genome sequence of the *P*. vulgaris G19833 accession [1] (http://www.phytozome.net/commonbean, accessed on 30 June 2021). In the filtering process, we included, for downstream analysis, only SNPs that showed minor allele frequency (MAF) >0.05; minimum quality >10; and mean read depth across all lines, ranging from 5 to 1000 (-maf 0.05-minQf 10-min-meanDP 5-max-meanDP 1000). VCFtools was used to estimate SNP and InDel statistics. The SNP density and transition/transversion ratio (Ts/Tv) were determined for nonoverlapping bins of 1 Mb. The ggplot2 package in R was used to construct graphs with the SNP density in each common bean chromosome.

### 4.4. Genetic and Population Structure

Population structure analysis was performed using the package LEA, an R package for landscape and ecological association studies. Parameters for population structure analysis included the number K from 2 to 10 groups and 10 iterations, as described by Frichot and François [68]. The delta k parameter used to calculate and define the best number of subpopulations was obtained by the LEA package. The R-based GAPIT package, a Genome Association and Prediction Integrated Tool, was used to calculate the VanRaden kinship matrix to develop an analysis of principal components (PCA).

For the phylogenetic analysis, we followed the methodology previously proposed by Ariani et al. [22]. First, SNPs located in repetitive regions were removed with VCFtools using the reference genome annotation of *P. vulgaris* [1]. Only variants within DNA coding sequences were used. Accessions that exhibited a minimum quality score of below 10, missing data, and heterozygous profiles (allele frequency higher than 0.05) were not included in the phylogenetic analyses. Missing SNPs were imputed using the Next Generation Sequencing Eclipse Plugin (NGSEP) [23]. A multiple sequence alignment file (in FASTA format), composed of SNPs extracted from each position for each evaluated genotype, was used to build the phylogenetic tree based on neighbor-joining (NJ) clustering methodology, considering the Kimura two-parameter model with 1000 bootstrap replicates. Phylogenetic analyses were conducted using MEGA7 software [69].

### 4.5. Genome-Environment Association Study (GEA)

Records of geographical coordinates were used in combination with bio-climatic variables, which were downloaded from the WorldClim database (http://www.worldclim.org, accessed on 30 June 2021) [70], to evaluate the habitat suitability of common bean accessions using maximum entropy modeling. Climatic variables were extracted using the Dismo package implemented in R software (www.r-project.org, accessed on 30 June 2021) (30 arc-second resolution). Bio-climatic variables were calculated based on monthly temperature and precipitation values, which are biologically more significant than simple average values because they represent annual trends such as seasonal effects and extreme weather conditions (Table 5).

Genome–environment associations were identified with TASSEL software [71]. Population structure (Q) analysis was performed by principal component analysis in TASSEL. The kinship matrix (developed using the identity by descent method implemented in TASSEL) was included in the association analysis to correct cryptic relatedness. The mixed linear model equation [72] used for association analysis was as follows:

(1) Υ=Xα+Pβ+Kμ+ε

where *Y* is the phenotype of one genotype; *X* is the fixed effect of a SNP; *P* is the fixed effect of the population structure; *K* is the random effect of the relative kinship; and ε is the error term, which is assumed to be normally distributed with a mean of zero. The percentage contribution of each SNP to the total phenotypic variation was calculated using the marker R^2^ (regression coefficient) through TASSEL and multiplied by 100 [73]. Moreover, a Manhattan plot was constructed and used to visualize the results. To improve the quality of the figures, we used R software to create Manhattan and quantile–quantile plots (Figures S3 and S4) with the package qqman [74]. The significance threshold for SNP–trait associations was the Bonferroni correction of *p* ≤ 0.001%, or smaller than the Bonferroni threshold (1.73 × 10^−6^ for α = 0.05 and 28,823 SNP markers).

### 4.6. Candidate-Gene Analysis

The putative functional annotation of each candidate gene was based on the descriptions available in the *P. vulgaris* reference genome v.1.0, available in Phytozome (https://phytozome-next.jgi.doe.gov/info/Pvulgaris_v2_1, accessed on 30 June 2021). Related to the bio-climatic variables, the predicted genes were identified using BLASTp in NCBI (National Center for Biotechnology Information; https://www.ncbi.nlm.nih.gov, accessed on 30 June 2021), and their putative homologs in *Arabidopsis thaliana* were observed. The *Arabidopsis thaliana* protein with the lowest E-value (<0.0) and highest identity (>40%) with each bean protein was considered to be a putative homolog and used to infer its molecular function.

## Figures and Tables

**Table 1:** Number of SNPs, the beginning, and the end in Mb for the 11 chromosomes of common bean accessions in Brazil.

Chromosome	Length (bp)	No. SNPs	Kbs/SNP	SNPs/Mb
Pv01	52,035,450	3269	15.91	62.82
Pv02	48,839,311	3651	13.37	74.75
Pv03	52,058,115	3262	15.95	62.66
Pv04	44,941,012	1788	25.13	39.78
Pv05	40,643,363	2269	17.91	55.82
Pv06	31,956,823	2304	13.87	72.09
Pv07	51,437,727	2266	22.69	44.05
Pv08	59,476,018	2276	26.13	38.26
Pv09	37,392,701	3310	11.29	88.51
Pv10	42,953,733	1660	25.87	38.64
Pv11	50,209,006	2768	18.13	55.12
Total	511,943,259	28,823	17.76	

**Table 2:** Transition and transversion for SNPs identified in 110 common bean accessions evaluated in this study.

Substitution	SNPs
Transition (Ts)	17,186
C/T	8551
A/G	8365
Transversion (Tv)	11,637
C/G	2504
A/T	3646
A/C	2685
G/T	2802
Ts/Tv ratio	1.581

**Table 3:** *Phaseolus vulgaris* chromosome (Chr), two most significant single nucleotide polymorphism (SNP) markers, trait, *p*-value, proportion of the phenotypic variation explained (R^2^), gene, and gene annotation of 110 common bean accessions.

Chr	SNP Marker ^a^	Trait ^b^	*p*-Value	R^2^	Gene	Gene Annotation
Pv01	S01_43225566	BIO2	3.50 × 10^−4^	13.5	*Phvul.001G169300*	Cation/H(+) antiporter 20
		BIO7	6.91 × 10^−5^	16.6		
		BIO18	8.74 × 10^−4^	13.0		
Pv02	S02_4770785	BIO1	8.96 × 10^−4^	11.7	*Phvul.002G051700*	No annotation
		BIO10	7.02 × 10^−4^	12.6		
		BIO13	2.47 × 10^−4^	17.7		
		BIO16	3.15 × 10^−4^	13.9		
	S02_5007350	BIO12	6.98 × 10^−4^	11.3	*Phvul.002G055050*	DNAJ heat shock *n*-terminal domain-containg protein
		BIO13	5.63 × 10^−4^	13.6		
Pv03	S03_13038972	BIO8	3.24 × 10^−4^	13.6	*Phvul.003G081100*	No annotation
		BI010	6.72 × 10^−4^	12.7		
		BIO12	7.68 × 10^−4^	13.1		
Pv04	S04_36754502	BIO16	4.15 × 10^−4^	14.5	*Phvul.004G111600*	Solute carrier family 13 member
		BIO19	1.37 × 10^−4^	14.0		
	S04_36771335	BIO6	1.37 × 10^−4^	13.5		
		BIO19	2.56 × 10^−4^	14.0		
Pv06	S06_18985703	BIO8	8.76 × 10^−4^	11.1	*Phvul.006G071300*	Basic region leucine zipper (bZIP_2)
Pv09	S09_30671474	BIO14	1.01 × 10^−6^	25.7	*Phvul.009G207800*	Leucine rich repeat proteins, some proteins contain F-box
		BIO17	1.11 × 10^−6^	25.4		
	S09_35475381	BIO2	6.50 × 10^−4^	11.9	*Phvul.009G241300*	Platz transcription factor family protein
		BIO3	4.57 × 10^−6^	22.8		
		BIO4	4.07 × 10^−8^	36.2		
		BIO6	1.69 × 10^−5^	21.1		
		BIO7	3.94 × 10^−5^	18.3		
		BIO9	1.32 × 10^−5^	21.0		
		BIO14	1.93 × 10^−7^	34.4		
		BIO15	1.65 × 10^−6^	25.2		
		BIO17	9.48 × 10^−8^	36.6		
Pv11	S11_9167649	BIO18	7.86 × 10^−4^	10.9	*Phvul.011G091600*	NTKL-binding protein 1
	S11_45868325	BIO3	4.20 × 10^−5^	16.2	*Phvul.011G182100*	No annotation
		BIO4	1.10 × 10^−5^	21.1		
		BIO9	1.18 × 10^−4^	15.5		

^(a)^ Marker ID contains the chromosome and the physical position of the polymorphism in the reference genome version 1.0; ^(b)^ BIO1: annual mean temperature; BIO2: mean diurnal range; BIO3: isothermality; BIO4: temperature seasonality; BIO5: max. temperature of warmest month; BIO6: min. temperature of coldest month; BIO7: temperature annual range; BIO8: mean temperature of wettest quarter; BIO9: mean temperature of driest quarter; BIO10: mean temperature of warmest quarter; BIO11: mean temperature of coldest quarter; BIO12: annual precipitation; BIO13: precipitation of wettest month; BIO14: precipitation of driest month; BIO15: precipitation seasonality (coefficient of variation); BIO16: precipitation of wettest quarter; BIO17: precipitation of driest quarter; BIO18: precipitation of warmest quarter; BIO19: precipitation of coldest quarter.

**Table 4:** Identification of the 110 common bean accessions from Brazil evaluated in this study.

Code	Common Name	State	City	Latitude	Longitude	Height (m)	Commercial Group	Gene Pool
BL_1	Brigida	PE	Recife	−8.05888	−34.880833	4	Carioca	M
BL_2	Coção	PE	Recife	−8.05888	−34.880833	4	Mantegão	A
BL_3	Bagajó	SE	Poço Verde	−10.707777	−38.182777	268	Mantegão	A
BL_4	Favita	PE	Garanhuns	−8.890277	−36.659444	842	Mantegão	A
BL_5	Canarinho	PE	Lajedo	−8.663888	−36.336666	661	Others	A
BL_6	Rosinha Claro	PE	Calçado	−8.741944	−36.333888	643	Rosinha	M
BL_7	Chita fina	PE	São João	−8.875833	−36.366944	716	Mantegão	A
BL_8	Jaula	PE	São João	−8.890277	−36.49277	842	Others	A
BL_9	Pintado	PE	Ibimirim	−8.540555	−37.690277	401	Mantegão	A
BL_10	Bolinha	PE	Lajedo	−8.540555	−36.32	532	Mulatinho	M
BL_11	Praia	SE	Poço Verde	−8.053888	−34.880833	268	White	A
BL_12	Camarão	PE	Calçado	−8.741944	−36.333888	643	Others	A
BL_13	BSF-1 Creme	PE	Belém do São Francisco	−8.753888	−38.963888	305	Mantegão	A
BL_14	BSF-2 Pingo de Ouro	PE	Belém do São Francisco	−8.053888	−34.88083	305	Carioca	M
BL_15	BSF-3 Fogo na serra	PE	Belém do São Francisco	−8.053888	−34.88083	305	Mantegão	A
BL_16	Brilhoso Mulatinho	PE	São João	−8.875833	−36.366944	716	Mulatinho	M
BL_18	Africano 4	PE	Recife	−8.05888	−34.880833	4	Mantegão	A
BL_19	IPA 1 Mulatinho	PE	Recife	−8.05888	−34.880833	4	Mulatinho	M
BL_20	IPA 7 Mulatinho	PE	Recife	−8.05888	−34.880833	4	Mulatinho	M
BL_24	Mulatinho de Cacho	PB	Arara	−6.827777	−35.757777	467	Mulatinho	M
BL_25	Mulatinho	PE	Jucati	−8.705833	−36.488888	820	Mulatinho	M
BL_26	Tachinha Mulatinho	PB	Arara	−6.827777	−35.757777	467	Mulatinho	M
BL_27	Mulatão	PE	Bezerros	−8.889999	−36.492777	470	Mantegão	A
BL_28	Bairasa 2	PE	Lajedo	−8.663888	−36.336666	661	Mulatinho	M
BL_29	Milagre de Sto Antônio	PE	Águas Belas	−9.110833	−37.122777	376	Mulatinho	M
BL_30	Flor Azul	PE	Águas Belas	−9.110833	−37.122777	376	Mulatinho	M
BL_31	Bico de ouro	PE	Águas Belas	−9.110833	−37.122777	376	Mulatinho	M
BL_33	Feijão mulatinho	PE	Caruaru	−8.282777	−35.975833	3	Mulatinho	M
BL_34	Feijão Laje	PB	São Miguel de Itaipu	−7.25	−35.21	45	Mulatinho	M
BL_35	Caiaminha	PE	Calçado	−8.741944	−36.333888	643	Rosinha	M
BL_41	CLPE7	PE	São João	−8.875833	−36.366944	716	Black	M
BL_50	CLPE 17	PE	Lajedo	−8.663888	−36.336666	661	Black	M
BL_57	Feijão Preto	PE	Lajedo	−8.663888	−36.336666	661	Black	M
BL_64	Feijão Colorido	PE	Arcoverde	−8.420833	−37.061388	663	Mantegão	A
BL_65	Feijão Preto	PE	Jucati	−8.705833	−36.488888	820	Black	M
BL_66	Feijão Carioca	PE	Caruaru	−8.282777	−35.975833	554	Carioca	M
BL_67	Feijão Carioca	PE	Sta Maria Cambucá	−7.84	−35.901944	494	Carioca	M
BL_68	Feijão Mulatinho	PE	Jupi	−8.711944	−36.415	782	Mulatinho	M
BL_69	Feijão Mulatinho	PE	Arcoverde	−8.420833	−37.061388	663	Mulatinho	M
BL_70	Feijão Carioca	PE	Vertentes	−7.902777	−35.987777	401	Carioca	M
BL_71	Feijão Mulatinho	PE	Arcoverde	−8.420833	−37.061388	663	Mulatinho	M
BL_72	Feijão Colorido	PE	Arcoverde	−8.420833	−37.061388	663	Others	A
BL_73	Feijão Colorido	PE	Arcoverde	−8.420833	−37.061388	663	Others	A
BL_74	Favita	PE	São João	−8.875833	−36.366944	716	Mantegão	A
BL_75	Favita	PE	São João	−8.875833	−36.366944	716	Mantegão	A
BL_76	Feijão Carioca	PE	São João	−8.875833	−36.366944	716	Carioca	M
BL_77	Enxofre	PE	São João	−8.875833	−36.366944	716	Others	A
BL_78	Favita	PE	São João	−8.875833	−36.366944	716	Mantegão	A
BL_79	Favita	PE	São João	−8.875833	−36.366944	716	Mantegão	A
BL_80	Feijão Preto	PE	São João	−8.875833	−36.366944	716	Black	M
BL_81	Feijão Preto	PE	São João	−8.875833	−36.366944	716	Black	M
BL_82	Feijão Carioca	PE	São João	−8.875833	−36.366944	716	Carioca	M
BL_83	Feijão Preto	PE	Lajedo	−8.663888	−36.336666	661	Black	M
BL_84	Feijão Preto	PE	Jucati	−8.705833	−36.488888	820	Black	M
BL_85	Feijão Carioca	PE	Jucati	−8.705833	−36.488888	820	Carioca	M
BL_86	Feijão Preto	PE	Jupi	−8.711944	−36.415	782	Black	M
BL_87	Feijão Carioca	PE	Arcoverde	−8.420833	−37.061388	663	Carioca	M
BL_88	Feijão Mulatinho	PE	São João	−8.875833	−36.366944	716	Mulatinho	M
BL_89	Feijão Branco	PE	Jupi	−8.711944	−36.415000	782	White	M
BL_90	Feijão Preto	PE	São João	−8.875833	−36.366944	716	Black	M
BL_91	Feijão Preto	PE	São João	−8.875833	−36.366944	716	Black	M
BL_92	Feijão Mulatinho	PE	Arcoverde	−8.420833	−37.061388	663	Mulatinho	M
BL_93	Feijão Colorido	PE	Casinha	−7.741111	−35.721111	390	Others	A
BL_94	Favita	PE	Lajedo	−8.663888	−36.336666	661	Mantegão	A
BL_95	Feijão Preto	PE	Calçado	−8.741944	−36.333888	643	Black	M
BL_96	Feijão Mulatinho	PE	Caruaru	−8.282777	−35.975833	554	Mulatinho	M
BL_97	Feijão Preto	PE	São Caetano	−8.325833	−36.142777	552	Black	M
BL_98	Feijão Colorido	PE	Caruaru	−8.282777	−35.975833	554	Others	A
BL_99	Feijão Preto	PE	Caruaru	−8.282777	−35.975833	554	Black	M
BL_100	Feijão Mulatinho	PE	Calçado	−8.741944	−36.333888	643	Mulatinho	M
BL_101	Feijão Mulatinho	PE	Riacho das Almas	−8.133888	−35.855833	407	Mulatinho	M
BL_102	Feijão Preto	PE	São Caetano	−8.325833	−36.142777	552	Black	M
BL_103	Feijão Preto	PE	São Caetano	−8.325833	−36.142777	552	Black	M
BL_104	Feijão Colorido	PE	Surubim	−7.831944	−35.755833	394	Others	M
BL_105	Feijão Mulatinho	PE	Santa Maria Cambucá	−7.84	−35.901944	494	Mulatinho	M
BL_106	BG-4	MT	Cáceres	−15.799572	−57.385088	180	Mulatinho	M
BL_107	BG-9	MT	Mirassol do Oeste	−15.583333	−57.979166	285	Mulatinho	M
BL_108	BG-13	MT	Cáceres	−15.731691	−57.351783	151	Mulatinho	M
BL_109	BG-17	MT	Cáceres	−16.261083	−58.292461	202	Mulatinho	M
BL_110	BG-18	MT	Cáceres	−16.251022	−58.294869	186	Mulatinho	M
BL_111	BG-23	MT	Cáceres	−15.998333	−57.481666	311	Mulatinho	M
BL_165	Pitanga	PR	Pitanga	−24.729	−51.721425	829	Others	A
BL_166	Corinthiano	PR	Loanda	−22.971027	−53.106013	344	Others	A
BL_168	Jalo Vermelho	PR	Capitão Leônidas Marques	−25.484708	−53.583041	380	Others	A
BL_170	Jalo Listras Pretas	PR	Nova Santa Rosa	−24.428666	−53.971819	480	Others	A
BL_172	BGF 20	PR	Terra Rica	−22.688794	−52.61755	381	Others	A
BL_181	MT 50G2	MT	Mirassol do Oeste	−15.496902	−58.044955	169	Mulatinho	M
BL_183	MT 55	MT	Mirassol do Oeste	−15.505158	−58.059069	172	Mulatinho	M
BL_184	MT 57G1	MT	Mirassol do Oeste	−15.521236	−58.049863	184	Mulatinho	M
BL_186	MT 62	MT	Mirassol do Oeste	−15.505158	−58.059069	172	Mulatinho	M
BL_187	MT 73G1	MT	Mirassol do Oeste	−15.538369	−58.047044	186	Mulatinho	M
BL_189	MT 79	MT	Mirassol do Oeste	−15.538369	−58.047044	186	Mulatinho	M
BL_194	MT 92	MT	Mirassol do Oeste	−15.571730	−58.028602	232	Mulatinho	M
BL_199	Enxofre	PE	Lajedo	−8.663888	−36.336666	661	Others	A
BL_215	Favita	PE	Arcoverde	−8.420833	−37.061388	663	Mantegão	A
BL_216	Feijão Carioca	PE	Santa Maria Cambucá	−7.84	−35.901944	494	Carioca	M
BL_220	Jalo Pintado 2	PR	Capitão Leônidas Marques	−25.484708	−53.583041	380	Others	A
BL_222	Gordo	PE	Lajedo	−8.663888	−36.336666	661	Others	A
BL_223	Feijão Mulatinho	PE	Águas Belas	−9.110833	−37.122777	376	Mulatinho	M
BL_224	Mulatinho	PE	São joão	−8.875833	−36.366944	716	Mulatinho	M
BL_225	Sempre Assim Branco	PE	Águas Belas	−9.110833	−37.122777	376	White	M
BL_226	CLPE3	PE	São João	−8.875833	−36.366944	716	Black	M
BL_227	CLPE4	PE	São João	−8.875833	−36.366944	716	Black	M
BL_228	CLPE8	PE	São João	−8.875833	−36.366944	716	Black	M
BL_229	CLPE10	PE	Caetés	−8.772777	−36.622777	849	Black	M
BL_230	CLPE11	PE	Jucati	−8.705833	−36.488888	820	Black	M
BL_231	CLPE12	PE	São João	−8.875833	−36.366944	716	Mulatinho	M
BL_232	CLPE14	PE	São João	−8.875833	−36.366944	716	Carioca	M
BL_233	CLPE15	PE	Caetés	−8.772777	−36.622777	849	Mulatinho	M
BL_234	Feijão Preto	PE	Calçado	−8.741944	−36.333888	643	Black	M

A—Andean, M—Mesoamerican, PE—Pernambuco, PB—Paraíba, SE—Sergipe, PR—Paraná, MT—Mato Grosso.

**Table 5:** Bio-climatic variables used for association analysis.

Abbreviation	Description of Bio-Climatic Variables
BIO1	Annual mean temperature
BIO2	Mean diurnal range (mean of monthly (max. temp–min. temp))
BIO3	Isothermality (BIO2/BIO7) (× 100)
BIO4	Temperature seasonality (standard deviation × 100)
BIO5	Max. temperature of warmest month
BIO6	Min. temperature of coldest month
BIO7	Temperature annual range (BIO5-BIO6)
BIO8	Mean temperature of wettest quarter
BIO9	Mean temperature of driest quarter
BIO10	Mean temperature of warmest quarter
BIO11	Mean temperature of coldest quarter
BIO12	Annual precipitation
BIO13	Precipitation of wettest month
BIO14	Precipitation of driest month
BIO15	Precipitation seasonality (coefficient of variation)
BIO16	Precipitation of wettest quarter
BIO17	Precipitation of driest quarter
BIO18	Precipitation of warmest quarter
BIO19	Precipitation of coldest quarter

## Data Availability

All data are presented within the article or in the Supplementary Materials.

## Supplementary Materials

The following are available online at https://www.mdpi.com/article/10.3390/plants10081572/s1**.** Supplementary material is available online for this article. Figure S1. Heatmap of pairwise kinship matrix values based on 28,823 SNPs on 110 common bean accessions, according to the VanRaden algorithm. The color histogram shows the distribution of the coefficients of the coancestry, and a stronger red color indicates the individuals who were more related to each other. The heatmap of the values in the kinship matrix was created using GAPIT. Figure S2. Density and distribution of SNPs in the genomic region of common bean accessions in Brazil. Figure S3. QQ plot showing the relationship between the expected and obtained −log10 (*p*-values) from GEA for BIO 1, BIO 2, BIO 3, BIO 4, BIO 6, BIO 7, BIO 8, BIO 9, and BIO10. Figure S4. QQ plot showing the relationship between the expected and obtained −log10 (*p*-values) from GEA for BIO12, BIO13, BIO14, BIO15, BIO16, BIO17, BIO18, and BIO19. Figure S5. Map of the region where common bean cultivars were collected in Pernambuco State. Table S1. Trait, *Phaseolus vulgaris* chromosome (Chr), single nucleotide polymorphism (SNP) markers, SNP position (bp), *p* value, -log (*p* value) of 110 common bean accessions. Table S2. Common bean accessions used for multiplexed sequencing and their respective barcodes.
