# Supplementary material for: Genome-Environment Association Analysis for Bio-Climatic Variables in Common Bean (Phaseolus vulgaris L.) from Brazil
Source: Plants (Basel). 2021 Jul 30;10(8):1572. doi: 10.3390/plants10081572 (PMC8399474; doi:10.3390/plants10081572)
Supplement: Supplementary file 1 [file plants-10-01572-s001.zip › plants-1304331-supp.pdf]

plants

Article

Genome-Environment Association Analysis for Bio-Climatic Variables in Common Bean (*Phaseolus vulgaris* L.) from Brazil

Júlio Cesar F. Elias [1], Maria Celeste Gonçalves-Vidigal [1,*], Andrea Ariani [2], Giseli Valentini [3], Maria da Conceição Martiniano-Souza [1], Mariana Vaz Bisneta [1] and Paul Gepts [4]

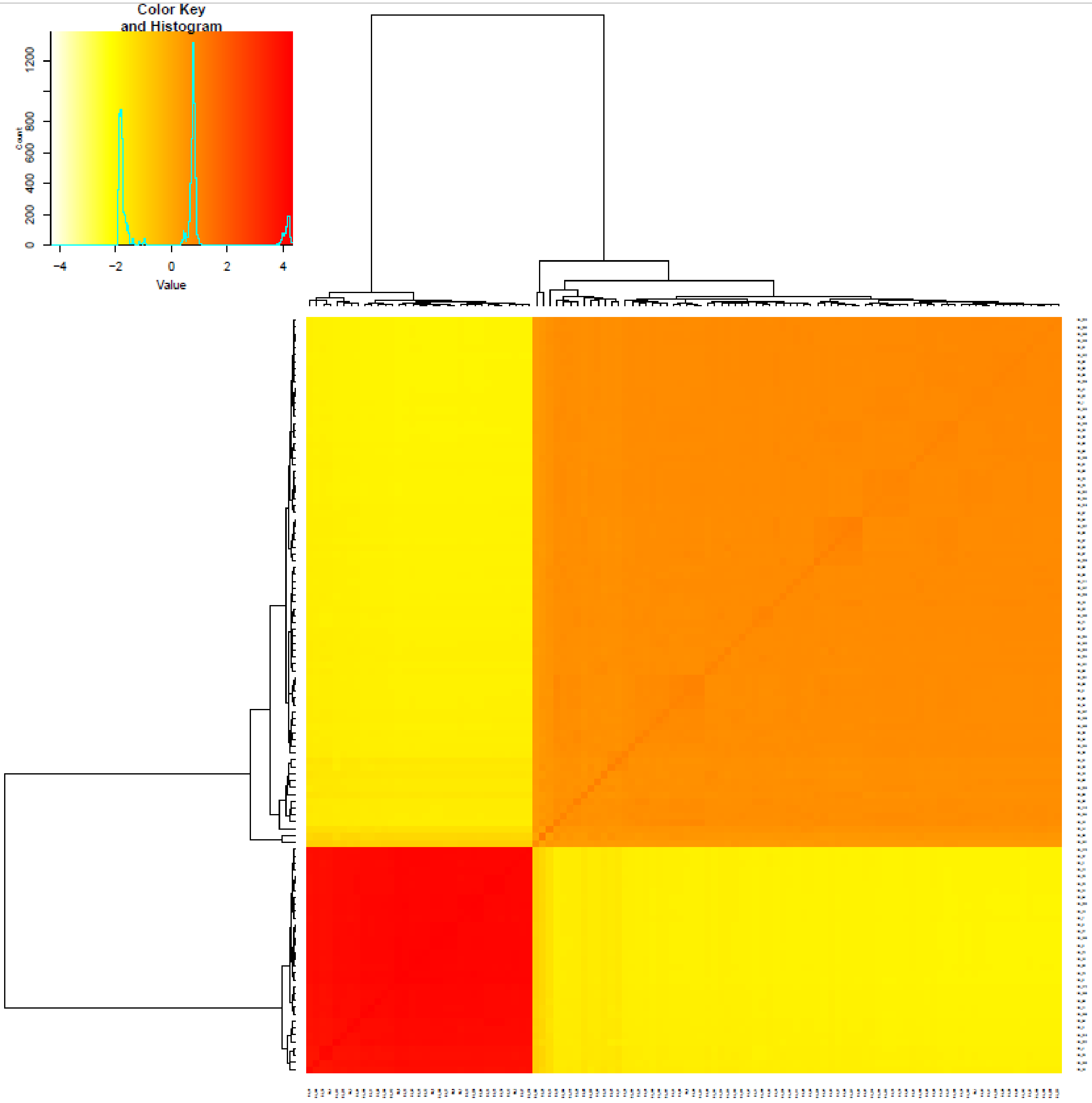

Figure S1. Heatmap of pairwise kinship matrix values based on 28,823 SNPs on 110 common bean accessions, according to the VanRaden algorithm. The color histogram shows the distribution of the coefficients of the coancestry, and a stronger

red color indicates the individuals who were more related to each other. The heatmap of the values in the kinship matrix was created using GAPIT.

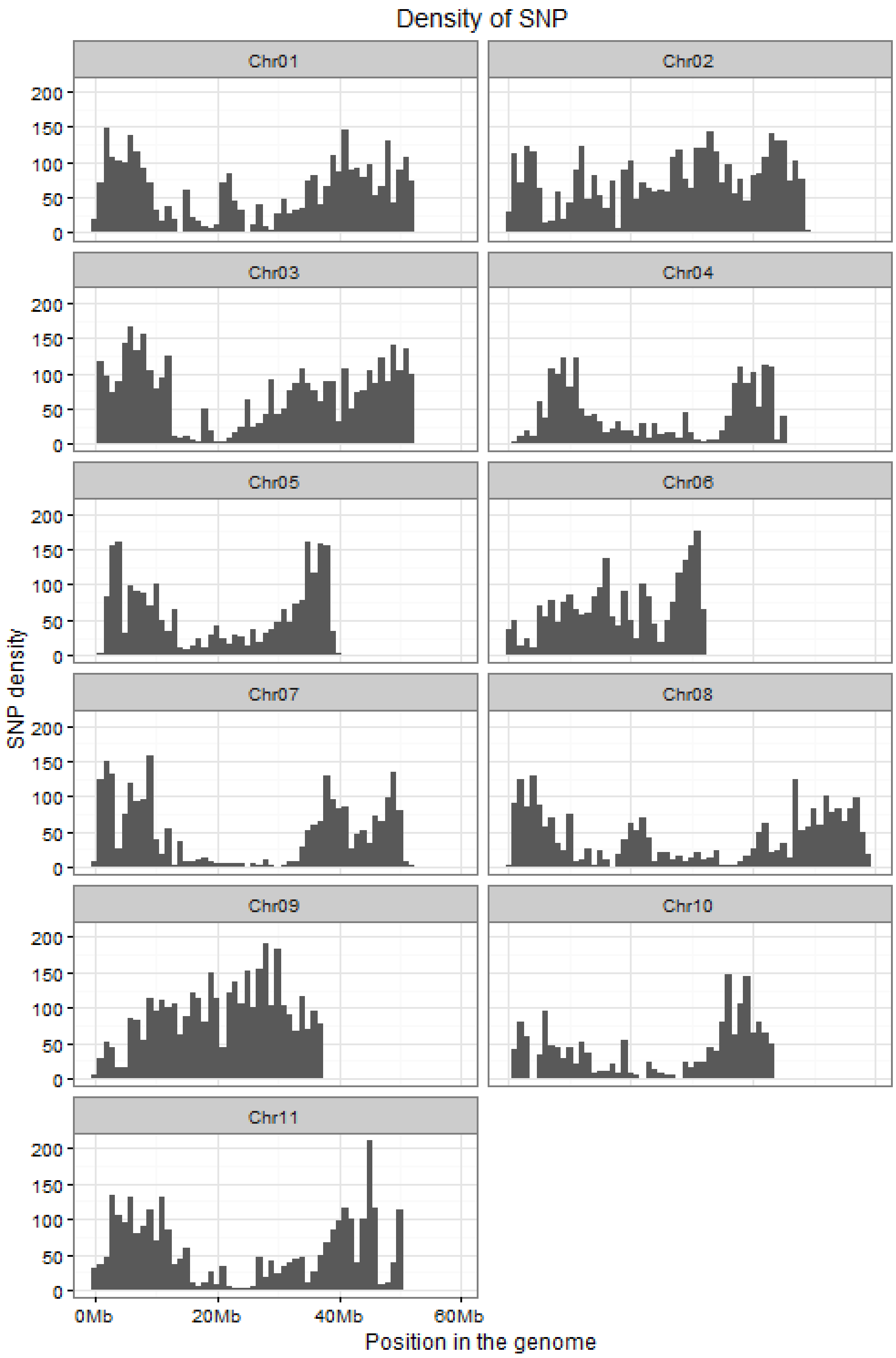

Figure S2. Density and distribution of SNPs in the genomic region of common bean accessions in Brazil.

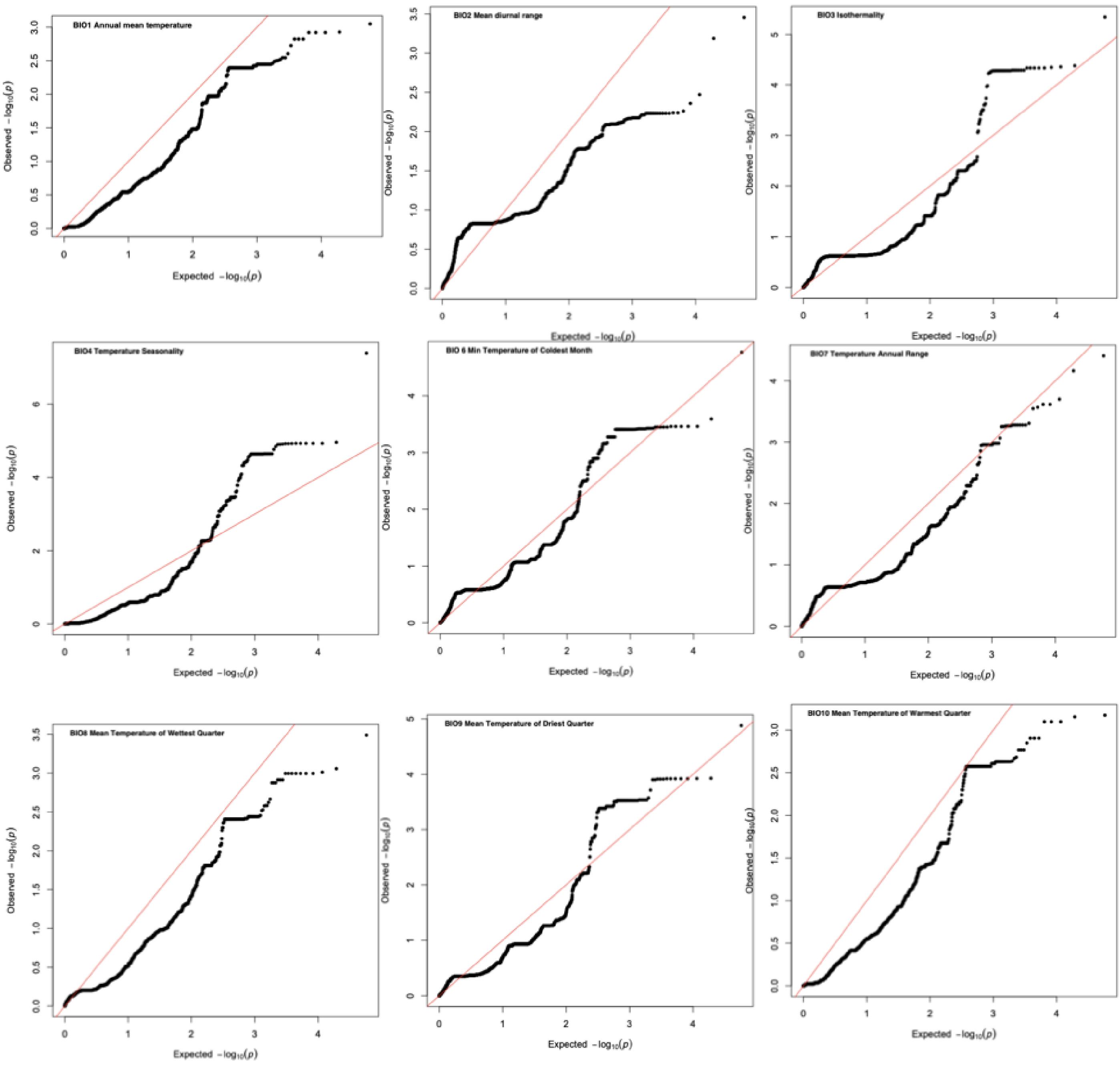

Figure S3. QQ plot showing the relationship between the expected and obtained −log10 (*p*-values) from GEA for BIO 1, BIO 2, BIO 3, BIO 4, BIO 6, BIO 7, BIO 8, BIO 9, and BIO10.

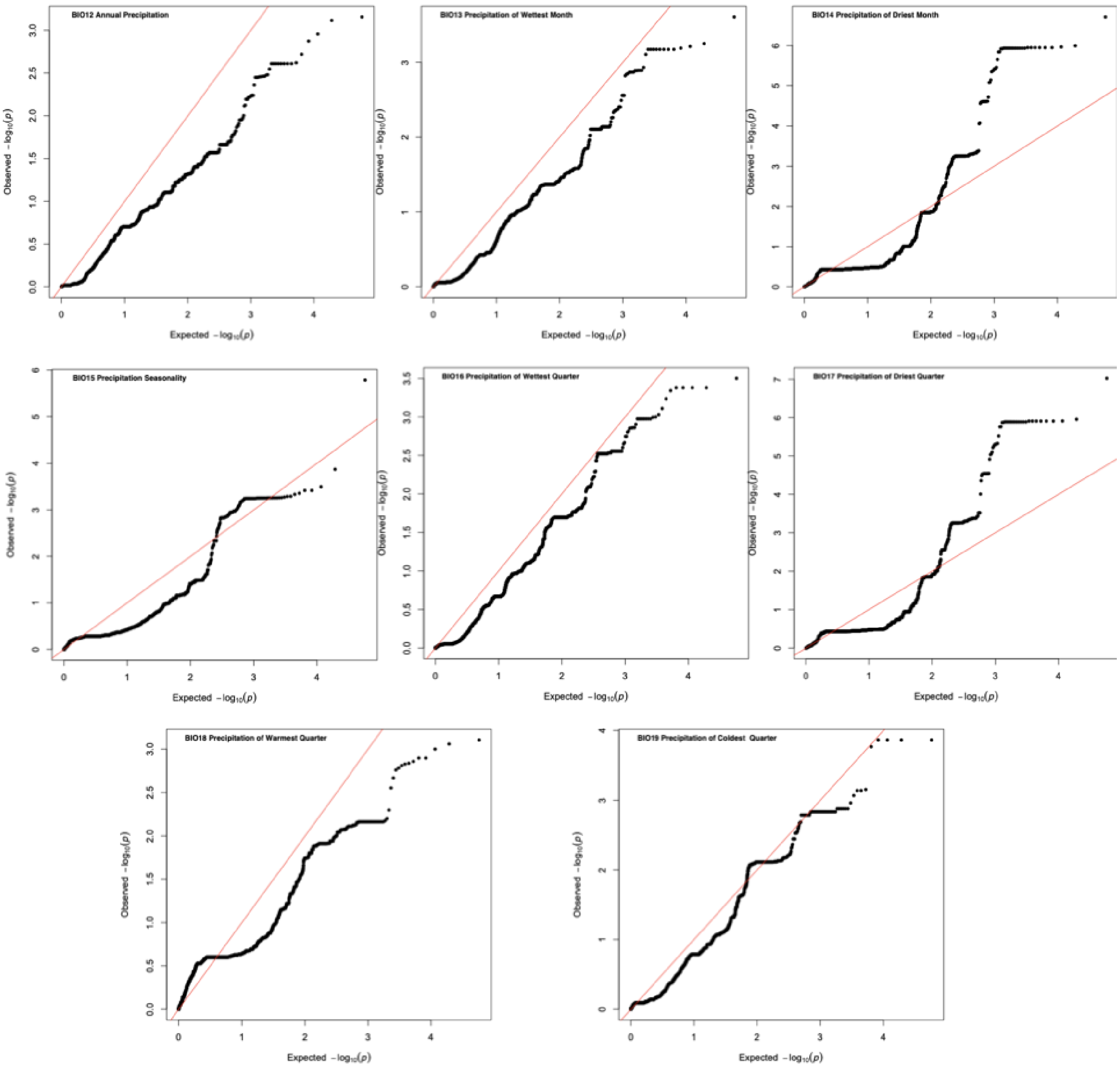

Figure S4. QQ plot showing the relationship between the expected and obtained −log10 (*p*-values) from GEA for BIO12, BIO13, BIO14, BIO15, BIO16, BIO17, BIO18, and BIO19.

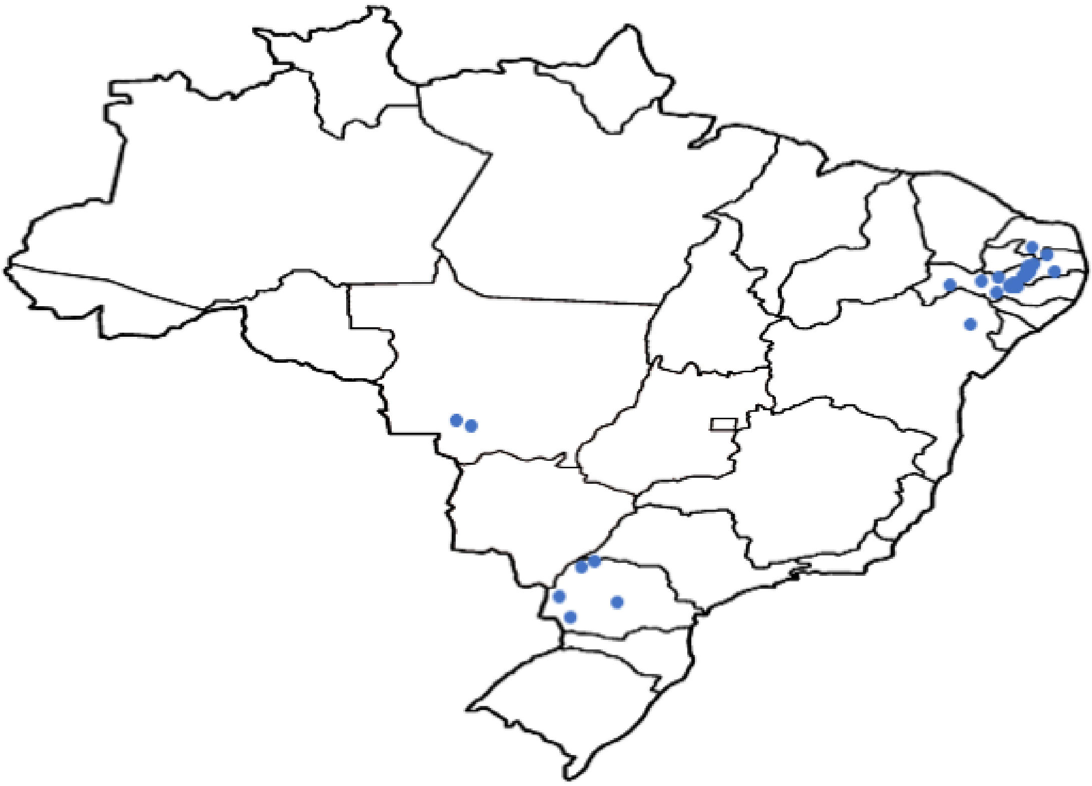

Figure S5. Map of the region where common bean cultivars were collected in Pernam-buco State.
