# Supplementary material for: Genome-Environment Association Analysis for Bio-Climatic Variables in Common Bean (Phaseolus vulgaris L.) from Brazil
Source: Plants (Basel). 2021 Jul 30;10(8):1572. doi: 10.3390/plants10081572 (PMC8399474; doi:10.3390/plants10081572)
Supplement: Supplementary file 1 [file plants-10-01572-s001.zip › S1 Table.pdf]

S1 Table Trait, *Phaseolus vulgaris* chromosome (Chr), Single Nucleotide Polymorfism (SNP) markers, SNP position (bp), *p* value, -log (*p* value) of 110 common bean accessions.

Trait	Chr	SNP marker	Position (bp)	p *value*	Log (*p*)
BIO1	2	S02_4770785	4770785	8.96E-04	3.05
BIO2	1	S01_43225566	43225566	3.50E-04	3.46
BIO2	9	S09_35475381	35475381	6.50E-04	3.19
BIO3	9	S09_35475381	35475381	4.57E-06	5.34
BIO3	11	S11_45868325	45868325	4.20E-05	4.38
BIO3	11	S11_45871518	45871518	4.42E-05	4.35
BIO3	9	S09_30671474	30671474	4.53E-05	4.34
BIO3	11	S11_46009051	46009051	4.66E-05	4.33
BIO3	11	S11_46026466	46026466	4.66E-05	4.33
BIO3	11	S11_46039222	46039222	4.66E-05	4.33
BIO3	11	S11_46086409	46086409	4.66E-05	4.33
BIO3	11	S11_46114790	46114790	4.66E-05	4.33
BIO3	2	S02_46562876	46562876	5.16E-05	4.29
BIO3	2	S02_46767945	46767945	5.16E-05	4.29
BIO3	2	S02_46774037	46774037	5.16E-05	4.29
BIO3	2	S02_46778765	46778765	5.16E-05	4.29
BIO3	9	S09_16894313	16894313	5.16E-05	4.29
BIO3	9	S09_17041160	17041160	5.16E-05	4.29
BIO3	11	S11_48564887	48564887	5.25E-05	4.28
BIO3	11	S11_48574427	48574427	5.25E-05	4.28
BIO3	11	S11_48590179	48590179	5.25E-05	4.28
BIO3	11	S11_48595485	48595485	5.25E-05	4.28
BIO3	11	S11_48673230	48673230	5.25E-05	4.28
BIO3	11	S11_48691105	48691105	5.25E-05	4.28
BIO3	11	S11_48717102	48717102	5.25E-05	4.28
BIO3	11	S11_48754682	48754682	5.25E-05	4.28
BIO3	11	S11_48769163	48769163	5.25E-05	4.28
BIO3	11	S11_48771356	48771356	5.25E-05	4.28
BIO3	11	S11_48790698	48790698	5.25E-05	4.28
BIO3	11	S11_48890465	48890465	5.25E-05	4.28
BIO3	11	S11_45901957	45901957	5.30E-05	4.28
BIO3	11	S11_45976959	45976959	5.30E-05	4.28
BIO3	11	S11_45979384	45979384	5.30E-05	4.28
BIO3	11	S11_48597386	48597386	5.42E-05	4.27
BIO3	2	S02_46636403	46636403	5.49E-05	4.26
BIO3	11	S11_45915796	45915796	5.81E-05	4.24
BIO3	11	S11_45927074	45927074	5.81E-05	4.24
BIO3	2	S02_46595538	46595538	5.99E-05	4.22
BIO3	9	S09_36970219	36970219	1.04E-04	3.98
BIO3	9	S09_36440025	36440025	1.08E-04	3.97
BIO3	1	S01_48656150	48656150	1.83E-04	3.74
BIO3	7	S07_44301353	44301353	2.36E-04	3.63
BIO3	11	S11_48916743	48916743	2.50E-04	3.60
BIO3	9	S09_4156404	4156404	2.51E-04	3.60

Trait	Chr	SNP marker	Position (bp)	p *value*	Log (*p*)
BIO3	5	S05_8091228	8091228	3.11E-04	3.51
BIO3	6	S06_13603868	13603868	3.11E-04	3.51
BIO3	10	S10_35224450	35224450	3.28E-04	3.48
BIO3	3	S03_1711893	1711893	3.41E-04	3.47
BIO3	2	S02_16724601	16724601	3.87E-04	3.41
BIO3	4	S04_19798525	19798525	4.87E-04	3.31
BIO3	11	S11_48535510	48535510	5.38E-04	3.27
BIO3	9	S09_35467033	35467033	5.95E-04	3.23
BIO3	3	S03_27967812	27967812	8.25E-04	3.08
BIO3	1	S01_43225566	43225566	8.78E-04	3.06
BIO4	9	S09_35475381	35475381	4.07E-08	7.39
BIO6	9	S09_35475381	35475381	1.69E-05	4.77
BIO6	4	S04_37237235	37237235	2.56E-04	3.59
BIO6	4	S04_41844379	41844379	3.45E-04	3.46
BIO6	4	S04_41850026	41850026	3.45E-04	3.46
BIO6	4	S04_41819945	41819945	3.46E-04	3.46
BIO6	4	S04_41865996	41865996	3.46E-04	3.46
BIO6	4	S04_41921556	41921556	3.46E-04	3.46
BIO6	11	S11_46009051	46009051	3.55E-04	3.45
BIO6	11	S11_46026466	46026466	3.55E-04	3.45
BIO6	11	S11_46039222	46039222	3.55E-04	3.45
BIO6	11	S11_46086409	46086409	3.55E-04	3.45
BIO6	11	S11_46114790	46114790	3.55E-04	3.45
BIO6	11	S11_45901957	45901957	3.72E-04	3.43
BIO6	11	S11_45871518	45871518	3.73E-04	3.43
BIO6	11	S11_45976959	45976959	3.73E-04	3.43
BIO6	11	S11_45979384	45979384	3.73E-04	3.43
BIO6	4	S04_37276206	37276206	3.81E-04	3.42
BIO6	4	S04_37323374	37323374	3.81E-04	3.42
BIO6	4	S04_37395494	37395494	3.81E-04	3.42
BIO6	4	S04_37402766	37402766	3.81E-04	3.42
BIO6	4	S04_37414095	37414095	3.81E-04	3.42
BIO6	4	S04_37419720	37419720	3.81E-04	3.42
BIO6	11	S11_45868325	45868325	3.84E-04	3.42
BIO6	11	S11_45927074	45927074	3.89E-04	3.41
BIO6	11	S11_45915796	45915796	3.90E-04	3.41
BIO6	4	S04_36860255	36860255	3.91E-04	3.41
BIO6	4	S04_36862905	36862905	3.91E-04	3.41
BIO6	4	S04_36898781	36898781	3.91E-04	3.41
BIO6	4	S04_36904123	36904123	3.91E-04	3.41
BIO6	4	S04_36923093	36923093	3.91E-04	3.41
BIO6	4	S04_37014401	37014401	3.91E-04	3.41
BIO6	4	S04_37044048	37044048	3.91E-04	3.41
BIO6	4	S04_37127964	37127964	3.91E-04	3.41
BIO6	4	S04_37171730	37171730	3.91E-04	3.41
BIO6	4	S04_37230271	37230271	3.91E-04	3.41
BIO6	4	S04_37233909	37233909	3.91E-04	3.41

Trait	Chr	SNP marker	Position (bp)	p *value*	Log (*p*)
BIO6	4	S04_37244713	37244713	3.91E-04	3.41
BIO6	4	S04_37261263	37261263	3.91E-04	3.41
BIO6	4	S04_37352176	37352176	3.91E-04	3.41
BIO6	4	S04_37389504	37389504	3.91E-04	3.41
BIO6	4	S04_37403736	37403736	3.91E-04	3.41
BIO6	4	S04_37404528	37404528	3.91E-04	3.41
BIO6	4	S04_37404856	37404856	3.91E-04	3.41
BIO6	4	S04_37412126	37412126	3.91E-04	3.41
BIO6	4	S04_37413884	37413884	3.91E-04	3.41
BIO6	4	S04_37426790	37426790	3.91E-04	3.41
BIO6	4	S04_37432423	37432423	3.91E-04	3.41
BIO6	4	S04_37452775	37452775	3.91E-04	3.41
BIO6	4	S04_37490566	37490566	3.91E-04	3.41
BIO6	4	S04_37501169	37501169	3.91E-04	3.41
BIO6	4	S04_36739231	36739231	5.31E-04	3.27
BIO6	4	S04_36792247	36792247	5.31E-04	3.27
BIO6	4	S04_36807595	36807595	5.31E-04	3.27
BIO6	4	S04_36858132	36858132	5.31E-04	3.27
BIO6	4	S04_36859433	36859433	5.31E-04	3.27
BIO6	4	S04_36860736	36860736	5.31E-04	3.27
BIO6	4	S04_36862528	36862528	5.31E-04	3.27
BIO6	4	S04_36867546	36867546	5.31E-04	3.27
BIO6	4	S04_36891598	36891598	5.31E-04	3.27
BIO6	4	S04_36915727	36915727	5.31E-04	3.27
BIO6	4	S04_36920211	36920211	5.31E-04	3.27
BIO6	4	S04_36967857	36967857	5.31E-04	3.27
BIO6	4	S04_36984541	36984541	5.31E-04	3.27
BIO6	4	S04_37002724	37002724	5.31E-04	3.27
BIO6	4	S04_37133177	37133177	5.31E-04	3.27
BIO6	4	S04_37165570	37165570	5.31E-04	3.27
BIO6	11	S11_1102750	1102750	6.66E-04	3.18
BIO6	11	S11_1085196	1085196	6.94E-04	3.16
BIO6	11	S11_1095899	1095899	6.94E-04	3.16
BIO6	11	S11_1103513	1103513	6.94E-04	3.16
BIO6	11	S11_1105090	1105090	6.94E-04	3.16
BIO6	11	S11_1208261	1208261	6.94E-04	3.16
BIO6	11	S11_1208868	1208868	6.94E-04	3.16
BIO6	11	S11_1212440	1212440	6.94E-04	3.16
BIO6	11	S11_1213505	1213505	6.94E-04	3.16
BIO6	11	S11_1222947	1222947	6.94E-04	3.16
BIO6	11	S11_1247658	1247658	6.94E-04	3.16
BIO6	11	S11_1262430	1262430	6.94E-04	3.16
BIO6	3	S03_27967812	27967812	7.09E-04	3.15
BIO6	10	S10_35224450	35224450	7.86E-04	3.10
BIO6	9	S09_35467033	35467033	8.64E-04	3.06
BIO6	7	S07_44301353	44301353	8.97E-04	3.05
BIO6	4	S04_36754502	36754502	9.02E-04	3.04

Trait	Chr	SNP marker	Position (bp)	p *value*	Log (*p*)
BIO6	4	S04_36771335	36771335	9.02E-04	3.04
BIO6	4	S04_36785530	36785530	9.02E-04	3.04
BIO6	4	S04_36799625	36799625	9.02E-04	3.04
BIO6	5	S05_8091228	8091228	9.51E-04	3.02
BIO6	6	S06_13603868	13603868	9.51E-04	3.02
BIO6	3	S03_1711893	1711893	0.001	3.00
BIO7	9	S09_35475381	35475381	3.94E-05	4.40
BIO7	1	S01_43225566	43225566	6.91E-05	4.16
BIO7	10	S10_35224450	35224450	2.00E-04	3.70
BIO7	5	S05_8091228	8091228	2.45E-04	3.61
BIO7	6	S06_13603868	13603868	2.45E-04	3.61
BIO7	7	S07_44301353	44301353	2.73E-04	3.56
BIO7	3	S03_1711893	1711893	2.87E-04	3.54
BIO7	4	S04_19798525	19798525	4.97E-04	3.30
BIO7	11	S11_45868325	45868325	5.29E-04	3.28
BIO7	11	S11_46009051	46009051	5.29E-04	3.28
BIO7	11	S11_46026466	46026466	5.29E-04	3.28
BIO7	11	S11_46039222	46039222	5.29E-04	3.28
BIO7	11	S11_46086409	46086409	5.29E-04	3.28
BIO7	11	S11_46114790	46114790	5.29E-04	3.28
BIO7	3	S03_27967812	27967812	5.45E-04	3.26
BIO7	11	S11_45901957	45901957	5.46E-04	3.26
BIO7	11	S11_45976959	45976959	5.50E-04	3.26
BIO7	11	S11_45979384	45979384	5.50E-04	3.26
BIO7	11	S11_45927074	45927074	5.58E-04	3.25
BIO7	11	S11_45915796	45915796	5.61E-04	3.25
BIO7	11	S11_45871518	45871518	5.63E-04	3.25
BIO7	9	S09_30671474	30671474	8.65E-04	3.06
BIO8	3	S03_13038972	13038972	3.24E-04	3.49
BIO8	6	S06_18985703	18985703	8.76E-04	3.06
BIO8	6	S06_18968432	18968432	9.73E-04	3.01
BIO9	9	S09_35475381	35475381	1.32E-05	4.88
BIO9	11	S11_45868325	45868325	1.18E-04	3.93
BIO9	11	S11_45871518	45871518	1.19E-04	3.92
BIO9	11	S11_46009051	46009051	1.20E-04	3.92
BIO9	11	S11_46026466	46026466	1.20E-04	3.92
BIO9	11	S11_46039222	46039222	1.20E-04	3.92
BIO9	11	S11_46086409	46086409	1.20E-04	3.92
BIO9	11	S11_46114790	46114790	1.20E-04	3.92
BIO9	11	S11_45901957	45901957	1.22E-04	3.91
BIO9	11	S11_45976959	45976959	1.22E-04	3.91
BIO9	11	S11_45979384	45979384	1.22E-04	3.91
BIO9	11	S11_45915796	45915796	1.25E-04	3.90
BIO9	11	S11_45927074	45927074	1.25E-04	3.90
BIO9	4	S04_37237235	37237235	1.92E-04	3.72
BIO9	9	S09_35467033	35467033	2.66E-04	3.58
BIO9	4	S04_37276206	37276206	2.89E-04	3.54

Trait	Chr	SNP marker	Position (bp)	p *value*	Log (*p*)
BIO9	4	S04_37323374	37323374	2.89E-04	3.54
BIO9	4	S04_37395494	37395494	2.89E-04	3.54
BIO9	4	S04_37402766	37402766	2.89E-04	3.54
BIO9	4	S04_37414095	37414095	2.89E-04	3.54
BIO9	4	S04_37419720	37419720	2.89E-04	3.54
BIO9	4	S04_36860255	36860255	2.96E-04	3.53
BIO9	4	S04_36862905	36862905	2.96E-04	3.53
BIO9	4	S04_36898781	36898781	2.96E-04	3.53
BIO9	4	S04_36904123	36904123	2.96E-04	3.53
BIO9	4	S04_36923093	36923093	2.96E-04	3.53
BIO9	4	S04_37014401	37014401	2.96E-04	3.53
BIO9	4	S04_37044048	37044048	2.96E-04	3.53
BIO9	4	S04_37127964	37127964	2.96E-04	3.53
BIO9	4	S04_37171730	37171730	2.96E-04	3.53
BIO9	4	S04_37230271	37230271	2.96E-04	3.53
BIO9	4	S04_37233909	37233909	2.96E-04	3.53
BIO9	4	S04_37244713	37244713	2.96E-04	3.53
BIO9	4	S04_37261263	37261263	2.96E-04	3.53
BIO9	4	S04_37352176	37352176	2.96E-04	3.53
BIO9	4	S04_37389504	37389504	2.96E-04	3.53
BIO9	4	S04_37403736	37403736	2.96E-04	3.53
BIO9	4	S04_37404528	37404528	2.96E-04	3.53
BIO9	4	S04_37404856	37404856	2.96E-04	3.53
BIO9	4	S04_37412126	37412126	2.96E-04	3.53
BIO9	4	S04_37413884	37413884	2.96E-04	3.53
BIO9	4	S04_37426790	37426790	2.96E-04	3.53
BIO9	4	S04_37432423	37432423	2.96E-04	3.53
BIO9	4	S04_37452775	37452775	2.96E-04	3.53
BIO9	4	S04_37490566	37490566	2.96E-04	3.53
BIO9	4	S04_37501169	37501169	2.96E-04	3.53
BIO9	4	S04_41844379	41844379	3.10E-04	3.51
BIO9	4	S04_41850026	41850026	3.10E-04	3.51
BIO9	4	S04_41819945	41819945	3.11E-04	3.51
BIO9	4	S04_41865996	41865996	3.11E-04	3.51
BIO9	4	S04_41921556	41921556	3.11E-04	3.51
BIO9	9	S09_30671474	30671474	3.56E-04	3.45
BIO9	4	S04_36739231	36739231	3.78E-04	3.42
BIO9	4	S04_36792247	36792247	3.78E-04	3.42
BIO9	4	S04_36807595	36807595	3.78E-04	3.42
BIO9	4	S04_36858132	36858132	3.78E-04	3.42
BIO9	4	S04_36859433	36859433	3.78E-04	3.42
BIO9	4	S04_36860736	36860736	3.78E-04	3.42
BIO9	4	S04_36862528	36862528	3.78E-04	3.42
BIO9	4	S04_36867546	36867546	3.78E-04	3.42
BIO9	4	S04_36891598	36891598	3.78E-04	3.42
BIO9	4	S04_36915727	36915727	3.78E-04	3.42
BIO9	4	S04_36920211	36920211	3.78E-04	3.42

Trait	Chr	SNP marker	Position (bp)	p *value*	Log (*p*)
BIO9	4	S04_36967857	36967857	3.78E-04	3.42
BIO9	4	S04_36984541	36984541	3.78E-04	3.42
BIO9	4	S04_37002724	37002724	3.78E-04	3.42
BIO9	4	S04_37133177	37133177	3.78E-04	3.42
BIO9	4	S04_37165570	37165570	3.78E-04	3.42
BIO9	11	S11_48597386	48597386	4.10E-04	3.39
BIO9	2	S02_46562876	46562876	4.13E-04	3.38
BIO9	2	S02_46767945	46767945	4.13E-04	3.38
BIO9	2	S02_46774037	46774037	4.13E-04	3.38
BIO9	2	S02_46778765	46778765	4.13E-04	3.38
BIO9	9	S09_16894313	16894313	4.13E-04	3.38
BIO9	9	S09_17041160	17041160	4.13E-04	3.38
BIO9	11	S11_48564887	48564887	4.13E-04	3.38
BIO9	11	S11_48574427	48574427	4.13E-04	3.38
BIO9	11	S11_48590179	48590179	4.13E-04	3.38
BIO9	11	S11_48595485	48595485	4.13E-04	3.38
BIO9	11	S11_48673230	48673230	4.13E-04	3.38
BIO9	11	S11_48691105	48691105	4.13E-04	3.38
BIO9	11	S11_48717102	48717102	4.13E-04	3.38
BIO9	11	S11_48754682	48754682	4.13E-04	3.38
BIO9	11	S11_48769163	48769163	4.13E-04	3.38
BIO9	11	S11_48771356	48771356	4.13E-04	3.38
BIO9	11	S11_48790698	48790698	4.13E-04	3.38
BIO9	11	S11_48890465	48890465	4.13E-04	3.38
BIO9	7	S07_44301353	44301353	4.20E-04	3.38
BIO9	10	S10_35224450	35224450	4.38E-04	3.36
BIO9	5	S05_8091228	8091228	4.80E-04	3.32
BIO9	6	S06_13603868	13603868	4.80E-04	3.32
BIO9	2	S02_46595538	46595538	4.83E-04	3.32
BIO9	2	S02_46636403	46636403	4.86E-04	3.31
BIO9	3	S03_1711893	1711893	4.95E-04	3.31
BIO9	4	S04_19798525	19798525	6.10E-04	3.21
BIO9	9	S09_4156404	4156404	8.13E-04	3.09
BIO9	3	S03_27967812	27967812	8.61E-04	3.06
BIO9	1	S01_48656150	48656150	8.64E-04	3.06
BIO9	9	S09_36970219	36970219	9.35E-04	3.03
BIO9	9	S09_36440025	36440025	9.63E-04	3.02
BI010	3	S03_13038972	13038972	6.72E-04	3.17
BIO10	2	S02_4770785	4770785	7.02E-04	3.15
BIO10	2	S02_23499179	23499179	7.99E-04	3.10
BIO10	2	S02_23522351	23522351	7.99E-04	3.10
BIO10	2	S02_23929739	23929739	7.99E-04	3.10
BIO12	2	S02_5007350	5007350	6.98E-04	3.16
BIO12	3	S03_13038972	13038972	7.68E-04	3.11
BIO13	2	S02_4770785	4770785	2.47E-04	3.61
BIO13	2	S02_5007350	5007350	5.63E-04	3.25
BIO13	2	S02_4757674	4757674	6.14E-04	3.21

Trait	Chr	SNP marker	Position (bp)	p *value*	Log (*p*)
BIO13	2	S02_6708073	6708073	6.46E-04	3.19
BIO13	2	S02_5727181	5727181	6.71E-04	3.17
BIO13	2	S02_5731880	5731880	6.71E-04	3.17
BIO13	2	S02_5738610	5738610	6.71E-04	3.17
BIO13	2	S02_5750826	5750826	6.71E-04	3.17
BIO13	2	S02_5765322	5765322	6.71E-04	3.17
BIO13	2	S02_5766404	5766404	6.71E-04	3.17
BIO13	2	S02_5817835	5817835	6.71E-04	3.17
BIO13	2	S02_5916263	5916263	6.71E-04	3.17
BIO13	2	S02_4963284	4963284	7.83E-04	3.11
BIO14	9	S09_35475381	35475381	1.93E-07	6.71
BIO14	9	S09_30671474	30671474	1.01E-06	6.00
BIO14	2	S02_46636403	46636403	1.08E-06	5.97
BIO14	2	S02_46562876	46562876	1.12E-06	5.95
BIO14	2	S02_46767945	46767945	1.12E-06	5.95
BIO14	2	S02_46774037	46774037	1.12E-06	5.95
BIO14	2	S02_46778765	46778765	1.12E-06	5.95
BIO14	9	S09_16894313	16894313	1.12E-06	5.95
BIO14	9	S09_17041160	17041160	1.12E-06	5.95
BIO14	11	S11_48564887	48564887	1.16E-06	5.94
BIO14	11	S11_48574427	48574427	1.16E-06	5.94
BIO14	11	S11_48590179	48590179	1.16E-06	5.94
BIO14	11	S11_48595485	48595485	1.16E-06	5.94
BIO14	11	S11_48673230	48673230	1.16E-06	5.94
BIO14	11	S11_48691105	48691105	1.16E-06	5.94
BIO14	11	S11_48717102	48717102	1.16E-06	5.94
BIO14	11	S11_48754682	48754682	1.16E-06	5.94
BIO14	11	S11_48769163	48769163	1.16E-06	5.94
BIO14	11	S11_48771356	48771356	1.16E-06	5.94
BIO14	11	S11_48790698	48790698	1.16E-06	5.94
BIO14	11	S11_48890465	48890465	1.16E-06	5.94
BIO14	2	S02_46595538	46595538	1.20E-06	5.92
BIO14	11	S11_48597386	48597386	1.20E-06	5.92
BIO14	9	S09_36970219	36970219	1.43E-06	5.84
BIO14	9	S09_36440025	36440025	1.44E-06	5.84
BIO15	9	S09_35475381	35475381	1.65E-06	5.78
BIO16	2	S02_4770785	4770785	3.15E-04	3.50
BIO16	4	S04_36754502	36754502	4.15E-04	3.38
BIO16	4	S04_36771335	36771335	4.15E-04	3.38
BIO16	4	S04_36785530	36785530	4.15E-04	3.38
BIO16	4	S04_36799625	36799625	4.15E-04	3.38
BIO16	5	S05_34603354	34603354	4.53E-04	3.34
BIO16	2	S02_5007350	5007350	5.79E-04	3.24
BIO16	2	S02_4757674	4757674	7.72E-04	3.11
BIO16	2	S02_4963284	4963284	9.35E-04	3.03
BIO16	2	S02_4766445	4766445	0.001	3.00
BIO17	9	S09_35475381	35475381	9.48E-08	7.02

Trait	Chr	SNP marker	Position (bp)	p *value*	Log (*p*)
BIO17	9	S09_30671474	30671474	1.11E-06	5.95
BIO17	2	S02_46636403	46636403	1.23E-06	5.91
BIO17	2	S02_46562876	46562876	1.24E-06	5.91
BIO17	2	S02_46767945	46767945	1.24E-06	5.91
BIO17	2	S02_46774037	46774037	1.24E-06	5.91
BIO17	2	S02_46778765	46778765	1.24E-06	5.91
BIO17	9	S09_16894313	16894313	1.24E-06	5.91
BIO17	9	S09_17041160	17041160	1.24E-06	5.91
BIO17	11	S11_48564887	48564887	1.30E-06	5.89
BIO17	11	S11_48574427	48574427	1.30E-06	5.89
BIO17	11	S11_48590179	48590179	1.30E-06	5.89
BIO17	11	S11_48595485	48595485	1.30E-06	5.89
BIO17	11	S11_48673230	48673230	1.30E-06	5.89
BIO17	11	S11_48691105	48691105	1.30E-06	5.89
BIO17	11	S11_48717102	48717102	1.30E-06	5.89
BIO17	11	S11_48754682	48754682	1.30E-06	5.89
BIO17	11	S11_48769163	48769163	1.30E-06	5.89
BIO17	11	S11_48771356	48771356	1.30E-06	5.89
BIO17	11	S11_48790698	48790698	1.30E-06	5.89
BIO17	11	S11_48890465	48890465	1.30E-06	5.89
BIO17	11	S11_48597386	48597386	1.34E-06	5.87
BIO17	2	S02_46595538	46595538	1.35E-06	5.87
BIO17	9	S09_36970219	36970219	1.74E-06	5.76
BIO17	9	S09_36440025	36440025	1.75E-06	5.76
BIO18	11	S11_9167649	9167649	7.86E-04	3.10
BIO18	1	S01_43225566	43225566	8.74E-04	3.06
BIO18	2	S02_11742975	11742975	0.001	3.00
BIO19	4	S04_36754502	36754502	1.37E-04	3.86
BIO19	4	S04_36771335	36771335	1.37E-04	3.86
BIO19	4	S04_36785530	36785530	1.37E-04	3.86
BIO19	4	S04_36799625	36799625	1.37E-04	3.86
BIO19	5	S05_34603354	34603354	1.71E-04	3.77
BIO19	11	S11_12127792	12127792	7.01E-04	3.15
BIO19	5	S05_34925867	34925867	7.24E-04	3.14
BIO19	11	S11_11008392	11008392	7.24E-04	3.14
BIO19	6	S06_30688446	30688446	8.55E-04	3.07
