# Supplementary material for: Genome-Environment Association Analysis for Bio-Climatic Variables in Common Bean (Phaseolus vulgaris L.) from Brazil
Source: Plants (Basel). 2021 Jul 30;10(8):1572. doi: 10.3390/plants10081572 (PMC8399474; doi:10.3390/plants10081572)
Supplement: Supplementary file 1 [file plants-10-01572-s001.zip › S2 Table.pdf]

S2 Table Common bean accessions used for multiplexed sequencing and their respective barcodes.

Code	Accession	Gene pool	Barcoded sequence
BL_1	Brigida	M	AACT
BL_2	Coção	A	CCAG
BL_3	Bagajó	A	TTGA
BL_4	Favita	A	GGTA
BL_5	Canarinho	A	ATTG
BL_6	Rosinha Claro	M	CGGT
BL_7	Chita fina	A	TGCG
BL_8	Jaula	A	GTAT
BL_9	Pintado	A	AACCT
BL_10	Bolinha	M	CCACG
BL_11	Praia	A	TATAT
BL_12	Camarão	A	GAGCG
BL_13	BSF-1 Creme	A	ACAAT
BL_14	BSF-2 Pingo de Ouro	M	CTCTG
BL_15	BSF-3 Fogo na serra	A	TCTGG
BL_16	Brilhoso Mulatinho	M	CGGCT
BL_18	Africano 4	A	ATTAG
BL_19	IPA 1 Mulatinho	M	TGCCG
BL_20	IPA 7 Mulatinho	M	GTGGT
BL_24	Mulatinho de Cacho	M	CACAA
BL_25	Mulatinho	M	GGAAG
BL_26	Tachinha Mulatinho	M	TCCTT
BL_27	Mulatão	A	AATGA
BL_28	Bairasa 2	M	ATGCA
BL_29	Milagre de Sto Antônio	M	CCTTA
BL_30	Flor Azul	M	CTAGA
BL_31	Bico de ouro	M	TGGAA
BL_33	Feijão mulatinho	M	GCCGA
BL_34	Feijão Laje	M	AGCTA
BL_35	Caiaminha	M	CATACG
BL_41	CLPE7	M	CCATCG
BL_50	CLPE17	M	ATGCCT
BL_57	Feijão Preto	M	TCAGGT
BL_64	Feijão Colorido	A	CAGGTT
BL_65	Feijão Preto	M	GTCCAT
BL_66	Feijão Carioca	M	TGATCA
BL_67	Feijão Carioca	M	TCTGAG
BL_68	Feijão Mulatinho	M	GTGACA
BL_69	Feijão Mulatinho	M	CGCAAT
BL_70	Feijão Carioca	M	GCGTAG
BL_71	Feijão Mulatinho	M	TAAGCCG
BL_72	Feijão Colorido	A	ATTCGCG
BL_73	Feijão Colorido	A	GGATCCG
BL_74	Favita	A	CACATCG
BL_75	Favita	A	ATGGCCG

Code	Accession	Gene pool	Barcoded sequence
BL_76	Feijão Carioca	M	TGACGCG
BL_77	Enxofre	A	GATACCG
BL_78	Favita	A	ACCTTCG
BL_79	Favita	A	CTAGGCG
BL_80	Feijão Preto	M	TATCTCG
BL_81	Feijão Preto	M	TGGACCG
BL_82	Feijão Carioca	M	GTCAGCG
BL_83	Feijão Preto	M	AGTGTCG
BL_84	Feijão Preto	M	GAGTTCG
BL_85	Feijão Carioca	M	CCTAGCG
BL_86	Feijão Preto	M	CCACTCG
BL_87	Feijão Carioca	M	TTCTACG
BL_88	Feijão Mulatinho	M	GAGGCCT
BL_89	Feijão Branco	M	CGTCACG
BL_90	Feijão Preto	M	ACACGCT
BL_91	Feijão Preto	M	ATCTCGG
BL_92	Feijão Mulatinho	M	GGAGGCT
BL_93	Feijão Colorido	A	TAGCCTG
BL_94	Favita	A	TTAATCG
BL_95	Feijão Preto	M	ACCTGTT
BL_96	Feijão Mulatinho	M	CGTACGG
BL_97	Feijão Preto	M	CCGGACT
BL_98	Feijão colorido	A	GACCTTG
BL_99	Feijão Preto	M	ATGTGCT
BL_100	Feijão Mulatinho	M	GTAGCGG
BL_101	Feijão Mulatinho	M	TGTAGCT
BL_102	Feijão Preto	M	CACTATG
BL_103	Feijão Preto	M	ACTCCGT
BL_104	Feijão Colorido	M	CAGGTGG
BL_105	Feijão Mulatinho	M	TGACATT
BL_106	BG-4	M	GCTTACG
BL_107	BG-9	M	TTCACTCG
BL_108	BG-13	M	GAGGTCCG
BL_109	BG-17	M	AGATGGCG
BL_110	BG-18	M	CCTACTCG
BL_111	BG-23	M	ATCCTCCG
BL_165	Pitanga	A	TGCCG
BL_166	Corinthiano	A	AATGA
BL_168	Jalo Vermelho	A	TATCGG
BL_170	Jalo Listras Pretas	A	ACGCTG
BL_172	BGF 20	A	GGTA
BL_181	MT 50G2	M	ATTG
BL_183	MT 55	M	ACGTG
BL_184	MT 57G1	M	CCTTA
BL_186	MT 62	M	AAGTCG
BL_187	MT 73G1	M	CGGATG
BL_189	MT 79	M	CTGGAG

Code	Accession	Gene pool	Barcoded sequence
BL_194	MT 92	M	CGGT
BL_199	Enxofre	A	TCGACG
BL_215	Favita	A	TAAGCCG
BL_216	Feijão Carioca	M	GTAT
BL_220	Jalo Pintado 2	A	AGTCCG
BL_222	Gordo	A	GCTATG
BL_223	Feijão Mulatinho	M	CAGGTT
BL_224	Mulatinho	M	ATTCGCG
BL_225	Sempre Assim Branco	M	GGATCCG
BL_226	CLPE3	M	CACATCG
BL_227	CLPE4	M	ATGGCCG
BL_228	CLPE8	M	TGACGCG
BL_229	CLPE10	M	GATACCG
BL_230	CLPE11	M	ACCTTCG
BL_231	CLPE12	M	CTAGGCG
BL_232	CLPE14	M	TATCTCG
BL_233	CLPE15	M	TGGACCG
BL_234	Feijão Preto	M	GTCAGCG

A= Andean, M=Mesoamerican
